# Cardiotoxicity of Uremic Toxins: A Driver of Cardiorenal Syndrome

**DOI:** 10.3390/toxins10090352

**Published:** 2018-09-01

**Authors:** Suree Lekawanvijit

**Affiliations:** Department of Pathology, Faculty of Medicine, Chiang Mai University, 110 Intawaroros Rd, Sribhoom, Chiang Mai 50200, Thailand; slekawan@hotmail.com; Tel.: +66-53-935442 (ext. 101); Fax: +66-53-935442 (ext. 102)

**Keywords:** uremic toxins, FGF23, protein-bound uremic toxin, indoxyl sulfate, p-cresyl sulfate, cardiotoxicity, cardiorenal syndrome

## Abstract

Cardiovascular disease (CVD) is highly prevalent in the setting of chronic kidney disease (CKD). Such coexistence of CVD and CKD—the so-called “cardiorenal or renocardiac syndrome”—contributes to exponentially increased risk of cardiovascular (CV) mortality. Uremic cardiomyopathy is a characteristic cardiac pathology commonly found in CKD. CKD patients are also predisposed to heart rhythm disorders especially atrial fibrillation. Traditional CV risk factors as well as known CKD-associated CV risk factors such as anemia are insufficient to explain CV complications in the CKD population. Accumulation of uremic retention solutes is a hallmark of impaired renal excretory function. Many of them have been considered inert solutes until their biological toxicity is unraveled and they become accepted as “uremic toxins”. Direct cardiotoxicity of uremic toxins has been increasingly demonstrated in recent years. This review offers a mechanistic insight into the pathological cardiac remodeling and dysfunction contributed by uremic toxins with a main focus on fibroblastic growth factor-23, an emerging toxin playing a central role in the chronic kidney disease–mineral bone disorder, and the two most investigated non-dialyzable protein-bound uremic toxins, indoxyl sulfate and p-cresyl sulfate. Potential therapeutic strategies that could address these toxins and their relevant mediated pathways since pre-dialysis stages are also discussed.

## 1. Introduction

Cardiovascular disease (CVD) is highly prevalent in the population with chronic kidney disease (CKD). The Chronic Renal Insufficiency Cohort study (*n* = 3885) demonstrated that 33% of CKD patients have concomitant CVD [1]. CKD with cardiovascular (CV) complications has been recognized as type 4 cardiorenal syndrome or renocardiac syndrome. In general population, renal impairment is an independent and strong CV risk factor. Even microalbuminuria without decline renal function is associated with a 2- to 4-fold increase in CV risk [2]. In patients with established CKD, progressive loss of renal function and CVD are two major outcomes; both are more related to the severity than the etiology of the kidney disease [3]. However, CKD patients are at greater risk of CV events or death than progression to end-stage renal disease (ESRD) [4]. Ninety per cent will suffer or die of CV complications before reaching ESRD [5]. In the dialysis population, CV mortality is responsible for approximately half of all deaths [6]. The risk of CV mortality is 10–30 times higher in the dialysis population than the general population and increases up to 500-fold in the young age group (25–34 years old) [7]. The major causes of cardiac deaths in CKD are sudden cardiac death, heart failure and myocardial infarction [8].

CKD itself is a progressive disease but symptomatically silent until approaching ESRD. Most patients usually experience uremic symptoms when structural pathology of both kidney and CV system is hardly reversible. CV pathology in the setting of CKD has its own specific characteristics.

First, cardiac structural and/or functional abnormalities in CKD, or the so-called “uremic cardiomyopathy,” are frequently present in the CKD population. This condition is characterized by left ventricular hypertrophy (LVH), striking cardiac fibrosis, intramyocardial arteriolar wall thickening and predominantly diastolic dysfunction, followed by systolic dysfunction at the late stage of overt heart failure. LVH is commonly used as a surrogate marker of uremic cardiomyopathy and a predictor of CV mortality. Notably, 75% of incipient dialysis patients have developed LVH [9], thereby being at high risk for cardiac death. In addition, CKD patients are predisposed to heart rhythm disorders with increased risk of sudden cardiac death [10,11]. Supraventricular arrhythmias, such as atrial fibrillation, are common; one-third of dialysis patients experience a supraventricular arrhythmia [12].

Second, ischemic heart disease accounts for only 15–25% of all cardiac deaths in the CKD population despite the high incidence of accelerated atherosclerosis and high fatality following myocardial infarction [13]. Half of CKD patients with angina pectoris or myocardial infarction are absent of significant luminal obstruction by coronary angiography [14]. On the contrary, non-obstructive vascular diseases such as vascular stiffness, calcification and ossification are highly prevalent in the CKD population and their pathogenesis is independent of hypertension [15,16]. This non-obstructive vascular pathology is a predictive marker of poor CV outcomes [17] and may at least in part explain the high proportion of ischemic heart disease without significant coronary atherosclerosis in the CKD population. 

Lastly, traditional CV risk factors such as hypertension, diabetes mellitus and hyperlipidemia appear to be insufficient to explain the CV complications, as described above, in the CKD population. CV mortality remains unacceptably high in this population despite the traditional CV risk factors being controlled [18]. Increasing evidence suggests that uremic toxins are potentially a non-traditional, CKD-specific CV risk factor. Among uremic toxins listed by the European Uremic Toxin Work Group [19,20,21], fibroblastic growth factor-23 (FGF23) and two protein-bound uremic toxins (PBUTs), indoxyl sulfate (IS) and p-cresyl sulfate (pCS) have been recently received much attention with regard to their adverse cardiovascular effects, demonstrated by a growing body of clinical and basic research in this field. 

## 2. Uremic Toxins as a CKD-Specific CV Risk Factor

In the dialysis population, CV morbidity and mortality is still unacceptably high [6]. Compared with renal transplant recipients, dialysis patients have significantly higher rates of all-cause mortality (6.3–8.2 vs 1.1–1.5 times greater than the normal population) [8] and a lower 5-year survival probability after the initiation of CVD (0.18 vs. 0.47) [6]. Progression of LVH is still observed in long-term hemodialysis patients [22,23], despite high blood pressure and anemia corrected [23]. The degree of cardiac fibrosis increases over time in hemodialysis patients but regresses over time after transplantation [24]. The duration of pre-transplantation hemodialysis is conversely correlated with the degree of cardiac function recovery following successful transplantation [25]. Furthermore, the prevalence of atrial fibrillation increases from 9–21% in pre-dialysis to 13–27% in long-term hemodialysis patients and the rate of sudden cardiac death falls in renal transplant recipients [10]. Such collective data have drawn attention to non-dialyzable uremic toxins as a possible CV risk factor in CKD patients. 

Based on the European Uremic Toxin Work Group (EUTox) [19,20,21], uremic retention compounds are defined by a ratio of uremic to normal concentration ratio > 1. A total number of 90 uremic retention compounds were included in the 2003 EUTox work [19] with additional 14 and 56 compounds published in 2007 [20] and 2012 [21], respectively. According to their physicochemical property, uremic retention compounds are generally classified into 3 groups. 

(1) Free water-soluble low-molecular weight or small water-soluble molecules (molecular weight (MW) < 500 Da) such as urea, creatinine and phosphorus. Molecules in this group are traditionally used as a marker of uremic retention and dialysis removal adequacy.

(2) Middle molecules (MW > 500 up to 32,000 Da) such as β_2_-microglobulin, parathyroid hormone (PTH) and FGF23. Removal of many middle molecules is limited by conventional hemodialysis. Better clearance has been demonstrated by using high-flux membrane or convection transport [26,27,28].

(3) Protein-bound molecules such as IS, pCS and homocysteine. Most are small (MW of < 500 Da) and bound to albumin (MW ~66,500 Da [29]). Retention of protein-bound molecules can competitively inhibit albumin binding of some drugs [30,31,32], thereby increasing risk of drug toxicity in uremic patients. Removal of these compounds is also severely limited by conventional or high-flux hemodialysis. Protein-leaking membrane and an additional adsorbent system have been proposed but further development is still required. 

Uremic retention compounds with harmful biological or biochemical activity are called ‘uremic toxins.’ Current conventional dialysis treatment ineffectively removes a number of uremic toxins, mainly due to the *large molecular size* and *high protein affinity* [26]. PBUTs appear to be the most problematic group, followed by middle molecules [33]. However, any accumulated uremic toxins, including small water-soluble molecules, can exert biological activity in the pre-dialysis stages for a long time, before reaching ESRD. In this context, uremic toxins are not only a consequence but also a driver of CKD progression and responsible for toxicity to various organs. In recent years, negative CV impact of a middle molecule FGF23 and two PBUTs, IS and pCS, has exponentially emerged but mostly with incompletely understood mechanisms. This review will discuss adverse CV effects of uremic toxins with a main focus on cardiotoxicity of FGF23 and IS and pCS. 

## 3. Cardiac Effects of Small Water-Soluble Molecules

Most small water-soluble uremic molecules have been considered inert thereby not being accepted as uremic toxins. However, apart from phosphorus, CV toxicity has been recently suggested for some small molecules, mostly based on a strong association with adverse CV outcomes from clinical studies.

Creatinine was demonstrated in vitro to suppress cardiac myocyte contractility and interfere with chloride channels long time ago [34]. However, clinical significance has never been reported. Similar to creatinine, urea had little adverse biological effects despite extensive study. Back in 1978, urea was once reported to reduce cardiac mechanical activity and increases cardiac oxygen consumption by lowering norepinephrine in isolated guinea pig hearts [35]. Until recently, a number of studies have suggested that urea may be biologically toxic to the kidney, intestinal barrier and vascular smooth muscle cells and in relation with insulin resistance state [36]. Some evidence indicates that urea may exert toxic effects through carbamylation, a post-translational modification, of proteins leading to diverse biological alterations such as changes related to atherogenesis [36]. Increased carbamylated proteins have been demonstrated in CKD [37] and are associated with adverse CV outcomes such as CV mortality, sudden cardiac death and death from heart failure [38]. 

Asymmetric dimethylarginine (ADMA) is an endogenous endothelial NO synthase inhibitor which induces endothelial dysfunction. A meta-analysis shows that elevated ADMA levels are associated with increased risk of CV events and all-cause mortality [39]. This association is strong in dialysis CKD patients [40]. ADMA-associated adverse CV outcomes are most likely related to vascular diseases that are contributed by oxidative stress-induced endothelial dysfuntion [41].

Uric acid is the end product of purines, freely filtered through glomeruli before 90% being reabsorbed at the proximal tubule, allowing 10% excreted in the urine [42]. Hyperuricemia is found in approximately half of incipient dialysis CKD patients. In peritoneal dialysis patients, each 1 mg/dL of increased uric acid levels is associated with 5% and 12% increased risk for all-cause and CV mortality, respectively [43]. Uric acid levels are also associated with an increased risk for incipient hypertension [44], new-onset heart failure [45] and atrial fibrillation [46] in diverse (mainly community-based) populations. However, study of a causative relationship between increased uric acid levels and adverse CV effects such as inflammation, oxidative stress, endothelial dysfunction, changes related to metabolic syndrome and abnormal activation of the renin-angiotensin-aldosterone system (RAAS) [47,48] has mainly focused on the vessel.

Trimethylamine-N-oxide (TMAO) is an intestinal microbiota-dependent metabolite of dietary choline [49,50]. Two meta-analyses have recently demonstrated that elevated TMAO levels were associated with a 23–67% increased risk of CV events and a 55–91% increased risk of all-cause mortality [51,52]. In preclinical studies, TMAO enhances renal dysfunction and tubulointerstitial fibrosis in a dose-dependent manner [53], as well as induces formation of aortic atherosclerotic plaque the size of which is positively correlated with TMAO levels in apoE^-^/^-^ mice [50]. In human, increased TMAO levels are associated with higher burden of coronary artery disease [50]. Similar to urea, ADMA and uric acid, direct cardiac effects of TMAO is still underinvestigated.

Phosphorus in the body is in either inorganic or organic phosphate. Approximately 85% of phosphate is in the bone, 10–15% in soft tissues and less than 1% in extra-cellular fluid [54]. About 90% of serum phosphate can be freely filtrated by the glomeruli while 10% is in protein-bound form. Serum phosphate levels are determined by renal excretion and dietary phosphorus intake. Hyperphosphatemia is one of the most problematic complications in CKD, manifesting as a hallmark of the chronic kidney disease—mineral bone disorder (CKD-MBD) [55]. Increased serum phosphate levels are associated with 20% increased risk of 1-year, all-cause mortality in incipient hemodialysis patients [56]. Abnormal phosphorus metabolism and its adverse CV effects will be discussed with FGF23 and PTH, another two key molecules involving in the CKD-MBD.

## 4. Cardiac Effects of Middle Molecules

According to publications from the EUTox, a number of middle molecules have been additionally listed in 2012 [21]. β_2_-microglobulin is a prototype of this group, commonly used as a representative marker of middle molecule retention and removal. Clearance of middle molecules is limited by conventional hemodialysis. Using higher permeability membranes (high-flux) or convection transport (hemofiltration/hemodiafiltration) have been shown to improve clearances of middle molecules such as β_2_-microglobulin, PTH and advanced glycosylation end products [26,27,28]. A reduction in middle molecule retention is independently associated with decreased risk of mortality [57].

Many middle molecules are implicated in CVD but mostly associated with atherogenesis. For example, oxidized LDL plays an important role in atherogenesis by enhancing foam cell generation, endothelial dysfunction, cell migration and apoptosis [58,59,60]. Advanced glycosylation end products are related to nitric oxide (NO) inactivation leading to vasoconstriction [26]. Also included in this group, tumor necrosis factor (TNF)-α and interleukin (IL)-6, key inflammatory cytokines involving in the progression of CVD and heart failure, can be upregulated by a number of uremic toxins. 

FGF23, a regulator of phosphate and vitamin D homeostasis, has recently emerged as a uremic toxin with cardiotoxicity.

### 4.1. Complex Interplay in the FGF23/Klotho/Phosphate/Vitamin D/PTH Axes 

FGF23, a bone derived phosphaturic hormone (30 kD glycoprotein), has emerged as a substance that plays a central role in the regulation of phosphate and vitamin D homeostasis. FGF23 is secreted in response to dietary phosphate intake and an increase in active 1,25-dihydroxyvitamin D levels. The physiological functions of FGF23 are generally mediated through activation of the FGF receptor 1/α-Klotho complex. Binding to the coreceptor Klotho enhances FGF23 affinity for FGF receptors. In the kidneys, FGF23 promotes phosphaturia by inhibiting phosphate reabsorption at proximal tubules via downregulating renal expression of sodium-dependent phosphate co-transporters and reduces 1,25-dihydroxyvitamin D production by inhibiting renal 1-α hydroxylase, CYP27B1 [61]. In an experimental study using a progressive CKD rat model treated with anti-FGF23, effects of FGF23 on phosphate excretion and reduced 1,25-dihydroxyvitamin D production were attenuated by neutralizing FGF23 levels thereby confirming a causative role of FGF23 in phosphate and vitamin D regulation [62]. In the parathyroid gland, FGF23 suppresses PTH secretion and PTH gene expression via the ERK1/2 mitogen-activated protein kinase (MAPK) pathway [63]. 

An increase in FGF23 levels have been consistently demonstrated in both experimental CKD models [62,64,65] and CKD patients [65,66,67]. 

In a study of 3,879 CKD patients, a significant increase in FGF23 and PHT levels and a decrease in 1,25-dihydroxyvitamin D levels were evident at stage 3 CKD when estimated glomerular filtration rate (eGFR) ≤ 59 mL/min/1.73 m^2^ [66]. While a gradual increase in serum phosphate was observed from eGFR ≤ 49 mL/min/1.73 m^2^ and overt hyperphosphatemia presented in late stage CKD when eGFR ≤ 29 mL/min/1.73 m^2^ [66]. This study also demonstrated that the number of participants with isolated FGF23 excess is > 6-fold more than isolated PTH excess despite the high prevalence of vitamin D deficiency and an eGFR threshold of a significant increase is higher for FGF23 comparing with PTH (57.8 59 vs 46.9 mL/min/1.73 m^2^) [66]. This suggests that FGF23 is elevated before PTH and is most likely to be the earliest marker of the CKD-MBD. The explanation of the slower increase of PTH than FGF23 may be due to the counterbalance effect of FGF23 on reducing PTH secretion. 

At present, the mechanism of an incipient elevation of FGF23 levels in early CKD is still unclear. Klotho deficiency has been proposed as an upstream step of FGF23 excess in the progression of the CKD-MBD however signs of Klotho deficiency such as elevated phosphate, 1,25-dihydroxyvitamin D and calcium levels are not present in early CKD. The other possibility is extra-skeletal sources such as an aberrant expression of FGF23 in the injured kidney tissue. In an acute kidney injury (AKI) mouse model induced by intraperitoneal folic acid injection, elevated plasma FGF23 levels were demonstrated after 1 h of AKI, independently of PTH and vitamin D signaling and dietary phosphate [68]. In the other study, plasma FGF23 levels significantly increased 2 h after an induction of unilateral ureter obstruction but renal FGF23 mRNA expression significantly increased later at 4 h [69]. The same study also showed an increase in plasma and renal mRNA expression of FGF23 in uremic rats induced by 5/6 nephrectomy for 8 weeks. However, there was no decrease in plasma FGF23 levels after removing the kidney rudiment (the remaining 1/6 kidney tissue) [69]. These findings suggest that renal FGF23 is unlikely to contribute to elevated plasma FGF23 levels, both in the setting of AKI and CKD. 

In early stage CKD, FGF23 maintains normal phosphate levels but reduces 1,25-dihydroxyvitamin D levels [62,66]. Decreased 1,25-dihydroxyvitamin D levels results in chronic parathyroid gland stimulation due to lack of inhibitory effects of 1,25-dihydroxyvitamin D on PTH gene transcription [70] and lack of inhibitory feedback to the parathyroid gland by subtle hypocalcemia [61]. 

However, Klotho expression declines with the progression of CKD [71,72]. This leads to a further compensatory increase in FGF-23 levels which fails to exert its phosphaturic and PTH-suppressing effects without Klotho [73], thereby enhancing hyperphosphatemia and overt hyperparathyroidism, conditions at high risk for CVD. On the contrary, excess FGF23 can exert Klotho-independent adverse effects on off-target organs for example pathologic cardiac remodeling, increased hepatic production of inflammatory cytokines and impaired leukocyte activity [74]. Such Klotho-independent off-target effects of FGF23 are mostly mediated by the activation of different FGF receptor isoforms and different cell signaling pathways from the physiologic action of FGF23 (discussed later). Interestingly, FGF-23 excess and Klotho deficiency per se, in isolation, have been demonstrated to adversely affect the heart. 

### 4.2. FGF23 Excess

A growing body of evidence based on both clinical and basic research supports cardiotoxicity of FGF23. Observational clinical studies show an association between FGF23 and LVH in both nondialysis [65] and dialysis [75] patients [76,77], CV events in incipient peritoneal dialysis patients [78] and 1-year, all-cause mortality in incipient hemodialysis patients [56]. The risk of death increases with levels of FGF23 [56]. Recently, a meta-analysis of 17 CKD cohorts and 17 general population cohorts showed that increased circulating FGF23 levels, almost up to 150 fold in the CKD population and especially high in dialysis-dependent patients, was associated with 48% increased risk for heart failure, 33% for myocardial infarction, 26% for stroke and 42% for CV mortality [79]. In one cohort study, an increased risk for decompensated heart failure in stage 2–4 CKD patients that is associated with FGF23 was independent of renal function [80]. When assessing the time course of CKD, FGF23 levels are associated with CV events only before the initiation of dialysis treatment; this association is independent of diabetes mellitus, prior CVD, eGFR, hemoglobin, use of RAAS blockade and all markers of the CKD-MBD (1,25-dihydroxyvitamin D, phosphate, PTH, calcium) [81]. This indicates that the negative CV impact of FGF23 is likely to develop since early stage CKD where FGF23 starts to increase. FGF23 also shows an independent association with accelerated CKD progression in early (stages 3 and lower) CKD patients [82] as well as with increased risk of coronary heart disease in the general population [83].

FGF23 can also be activated following cardiac injury per se, independent of renal function. Elevation of circulating FGF23 levels are demonstrated in both animal heart failure model [84,85] and in patients with acute decompensated heart failure [86,87] or myocardial infarction [88] and are associated with poor prognosis [87]. In addition, expression of cardiac FGF23 is also induced by CVD such as in myocardial infarction [84,85]. In patients with early CKD, FGF23 levels are significantly higher in patients with heart failure than without heart failure [89]. With its biological toxicity to the cardiovascular system and the kidneys, FGF23 appears to be one of the key drivers in the cardio-renal connection that accelerates the progression of CKD and heart failure as well as the progression of cardiorenal syndrome.

Experimental studies demonstrated direct detrimental cardiac effects of FGF23 including LVH [65], cardiac fibrosis [84], cardiac mechanical dysfunction [90] and cardiac arrhythmias [91]. Of note, these off-target effects of FGF23 are Klotho-independent.

#### 4.2.1. Left Ventricular Hypertrophy

Associations between high FGF23 levels and increased LV mass as well as increased risk of LVH have been demonstrated in a community-based elderly (≥65 years) population (*n* = 2255) with a CKD prevalence of 32% [76]. Analysis based on CKD status shows that such associations are stronger in participants with CKD than without CKD [76,77].

A combined clinical and experimental study has demonstrated a mechanistic insight of FGF23-induced LVH for the first time. This study found that FGF23 levels were independently associated with LVH in a non-dialysis dependent CKD population (*n* = 3070) and induced LVH, both in vitro and in vivo, through the Klotho-independent FGF receptor activation of the phospholipase Cγ/calcineurin/nuclear factor of activated T cell signaling pathway, without activation of PI3K-Akt, a mediator implicated in physiological rather than pathological hypertrophy [65]. This activated signaling pathway is different from that responsible for physiologic function of FGF23 which is the FGFR1-Klotho complex/RAS/ERK1/2 MAPK pathway [63,74]. Of note, FGF23-induced LVH through the activation of FGF receptor in this study was confirmed by using a pan FGF receptor blocker [65]. Later studies further illustrated that FGF23-induced cardiomyocyte and LV hypertrophy were mediated by activation of FGF receptor 4 because these effects were attenuated by blockade of FGF receptor 4 [92,93] and *FGFR4* deficient animals did not develop LVH [93]. Interestingly, high phosphate diet also stimulated FGF23-induced cardiac hypertrophy and fibrosis in wild-type C57BL/6J mice that was reversible after switching to regular diet [93]. 

Neutralization of FGF23 by a monoclonal FGF23 antibody has been demonstrated in a rat model of CKD-BMD [94]. Despite an improvement of hyperparathyroidism, FGF23 did not change renal function and pathology and cardiac mRNA expression of pro-hypertrophic markers including skeletal muscle α-actin, myosin heavy chain (MHC) and atrial natriuretic peptide (ANP). On the contrary, treatment with FGF23 antibody further increased serum phosphate levels, consistent with no change in renal expression of sodium-phosphate co-transporters and Klotho genes and promoted aortic calcification [94]. High dose FGF23 antibody resulted in decline in renal function and 50% mortality rate.

Nevertheless, blockade of FGF receptors has been shown to improve LVH, myocardial fibrosis and cardiac function and to reduce cardiac mRNA expression of pro-hypertrophic markers (α-MHC, β-MHC, ANP and B-type natriuretic peptide (BNP)) in 5/6 nephrectomy rats with FGF23 excess [95]. While there was no changes in renal function, FGF23 levels, phosphate levels and blood pressure. Given that FGF receptor 4 is responsible for the pathologic functions of FGF23, selective FGF receptor 4 blockers might be more specific to target FGF23-induced cardiac hypertrophy and do not interfere with the physiologic functions of FGF23. 

The off-target detrimental biological activity of FGF23 excess potentially starts early therefore it should be addressed from pre-dialysis CKD. In addition, FGF23 excess of which the levels can be extremely high in patients with end-stage renal disease, may be not easily removed by dialysis even by convection technique [96,97]. This supports the evidence of LVH progression in long-term hemodialysis patients [22,23]. 

#### 4.2.2. Cardiac Fibrosis

Evidence of FGF23 stimulating cardiac fibrosis has recently demonstrated in cultured cardiac fibroblasts and in mice models of myocardial infarction and cardiac ischemic-reperfusion injury [84]. In this study, FGF23 promoted cell proliferation, myocardial fibrosis and upregulation of collagen I and III as well as β-catenin, transforming growth factor (TGF)-β and FGF receptor 4. Inhibition of β-catenin attenuates FGF23-induced cell proliferation, myocardial fibrosis and TGF- β upregulation [84]. Such findings suggest that the cardiac pro-fibrotic effect of FGF23 is likely be mediated through TGF-β and β-catenin pathways via activation of FGF receptor 4. Likewise, FGF23, in the absence of Klotho, exhibits a renal pro-fibrotic effect by enhancing gene expression of TGF-β via activation of FGF receptor 4 in fibroblasts derived from injured fibrotic kidneys [98]. The crosstalk between the β-catenin pathway and the TGF-β pathway has been demonstrated to plays a role in renal fibrosis [99], however this is still uncertain in cardiac fibrosis.

#### 4.2.3. Cardiac Mechanical Dysfunction 

In cardiomyocytes isolated from 5/6 nephrectomy mice with FGF23 injection, impaired calcium handling determined by delay in cytosolic calcium rise during systole and delay in the decay of cytosolic calcium during diastole was demonstrated, suggesting role of FGF23 in myocardial contractile dysfunction [64]. A combined in vitro, ex vivo and in vivo study showed that FGF23 increased intracellular Ca^2+^ in isolated cardiomyocytes treated with FGF23 and increased contractility of mice cardiac muscle strips [90]. On the contrary, the in vivo experiment of the same study showed impaired contractility by a reduction in fractional shortening and ejection fraction in CKD (Col4a3^−^/^−^) mice with FGF23 excess however calcium flux was not investigated. The authors suggested that FGF23 acutely increases intracellular Ca^2+^ with increased myocardial contractility but in long term, this may lead to systolic dysfunction. The effect of FGF23 on increases contractility of isolated cardiac muscle strips has been demonstrated to be inhibited by blockade of FGF receptors 1 [90] and 4 [93]. Such impaired contractility effect may at least in part support clinical findings of high FGF23 levels in patients with systolic heart failure and of FGF23-associated LV dysfunction in the absence of LVH [100].

#### 4.2.4. Cardiac Arrhythmias

CKD patients are predisposed to heart rhythm disorders such as atrial fibrillation, ventricular arrhythmias and sudden cardiac death, one of the most common causes of death. In stage 5 CKD patients, FGF23 levels are independently associated with decreased heart rate variability, a predictive marker of cardiac arrhythmias [67]. A strong independent association between FGF23 levels and atrial fibrillation have been demonstrated in both CKD [101] and non-CKD [100,102] populations. The mechanism of FGF23-induced atrial fibrillation has been demonstrated in pulmonary vein cardiomyocytes of which the ectopic activity is the most important trigger of atrial fibrillation [91]. Treatment with FGF23, pulmonary vein cardiomyocytes showed a faster beat rate, larger late sodium currents and calcium transient (together with larger sodium-calcium exchanger and L-type calcium currents) and a greater increase in mitochondrial reactive oxygen species (ROS) expression than non-treated cells; all of these effects are inhibited by an inhibitor of late sodium currents. The FGF23 effect on late sodium currents was prevented by inhibition of protein kinase C and calcium/calmodulin-dependent protein kinase II (CaMK II) [91]. CaMK II-enhanced late sodium currents lead to calcium overload [103], that may subsequently trigger arrhythmogenesis. Interestingly, the phospholipase Cγ/calcineurin/nuclear factor of activated T cell signaling pathway that mediates FGF23-induced LVH [65] is unlikely to be involved in the FGF23-induced calcium dysregulation since there is no change in calcineurin expression in FGF23-treated pulmonary vein cardiomyocytes [91].

#### 4.2.5. Potential Therapeutic Strategies for FGF23-Induced Cardiotoxicity

***Direct strategies*** include FGF23 neutralization and FGF receptor blockade. Anti-FGF23 neutralizing antibodies and FGF receptor inhibition have been demonstrated to improve survival and host defense by enhancing neutrophil recruitment and bacterial clearance in 5/6 nephrectomy mice after inducing pneumonia [104]. However, only FGF receptor blockade but not anti-FGF23 antibodies [94], shows beneficial effects on attenuating FGF23-induced LVH [65]. According to the experimental studies, selective blockade of FGF receptor 4 is recommended for treatment of cardiac hypertrophy and fibrosis induced by FGF23 because this process is selectively mediated via FGF receptor 4 activation [84,93]. 

***Indirect strategies*** include vitamin D supplement and low phosphate diet. In 5/6 nephrectomy rats with elevated cardiac FGF23 expresison, treatment with 1,25-dihydroxyvitamin D attenuates LVH, cardiac FGF receptor 4 expression and cardiac calcineurin/ nuclear factor of activated T cell activation [105]. Other than the inhibitory effect on cardiac FGF receptor 4 expression, an anti-cardiac hypertrophic effect of vitamin D has also been demonstrated in isolated cardiac myocytes [106], in Dahl rats, an animal model of LVH and vitamin D deficiency [107] and in hemodialysis patients [108,109]. This cardioprotective effect appears to be due at least in part to a PTH-lowering effect of vitamin D [108,109]. Interestingly, dietary phosphate restriction, a strategy normally used in CKD patients with hyperphosphatemia, can reduce FGF23 levels [110] thereby potentially preventing cardiotoxicity of FGF23. A reversal of FGF23-induced cardiac remodeling has been demonstrated in an animal model after switching from high phosphate diet to normal diet, as previously mentioned [93].

### 4.3. Klotho Deficiency

Although, the off-target cardiac effects of FGF23 are mainly attributable by a compensatory increase in FGF23 levels secondary to a state of Klotho deficiency in progressive CKD, some evidence suggests that Klotho deficiency itself also adversely affects the heart. An experimental study demonstrated more severe cardiac hypertrophy, fibrosis and dysfunction in heterozygous Klotho-deficient CKD than in wild-type CKD mice [110]. Intravenous delivery of a transgene encoding soluble Klotho ameliorated cardiac hypertrophy in Klotho-deficient CKD mice without changes in serum FGF23 and phosphate levels. While normalization of serum FGF23 and phosphate levels by dietary phosphate restriction and blood pressure with antihypertensive drugs did not reverse cardiac hypertrophy in Klotho-deficient CKD mice. Collectively, this suggests a cardioprotective effect of Klotho in the setting of CKD that is independent of FGF23 excess, hyperphosphatemia and hypertension.

### 4.4. Hyperphosphatemia

Abnormal calcium-phosphorus metabolism is associated with the progression of CKD [111] as well as development of CV complications. The kidney, bone and parathyroid gland act in concert in regulation of calcium and phosphorus homeostasis, however CKD appears to be the most common cause of disturbance. Mineral disorders in the CKD-MBD, owing largely to hyperphosphatemia, are independently associated with mortality and morbidity associated with CVD and fracture in hemodialysis patients [112]. Hyperphosphatemia usually present in advance stage CKD [112]. High levels of phosphorus and calcium-phosphorus product are associated with CV events and all-cause mortality in the hemodialysis population (*n* = 14,829) [113]. In fact, such associations are not only present in the CKD population but also in the general population [114,115]. 

Hyperphosphatemia is linked to high prevalence of vascular calcification in hemodialysis patients [116] and cardiac fibrosis and intramyocardial arterial wall thickening in a CKD model fed with high phosphorus diet [117]. Progression of coronary calcification is predictive of cardiac death and myocardial infarction in pre-dialysis CKD patients [118]. Therefore, hyperphosphatemia-associated vascular calcification may be at least in part responsible for deaths due to ischemic heart disease without significant coronary obstruction in the CKD population. 

Although, most experimental studies on adverse CV effects of phosphate mostly focus on vascular effects, there have been some studies demonstrating cardiac injury contributed by hyperphosphatemia. Phosphate exhibits an apoptotic effect in human cardiac myocytes by inducing cell death, predominantly of apoptotic type with lesser necrotic type, upregulating expression of cleaved Caspase-3 proapoptotic protein and inhibiting cell proliferation in a time– and concentration–dependent manner; all are prevented by blocking intracellular phosphate uptake [119]. Consistent with these findings, a study using cultured cardiomyoblast cells showed that phosphate-induced apoptosis was accompanied by increased autophagosome formation and the expression of autophagy-related proteins, as well as increased adenosine monophosphate (AMP)-activated protein kinase and UNC-51-like kinase 1 (ULK1) protein expression [120]. Apoptosis induced by phosphate was neutralized by autophagy inhibition, suggesting that phosphate-induced apoptosis is mediated by autophagy and activation of the AMP-activated protein kinase and ULK1 pathway.

In 5/6 nephrectomy CKD rats fed with a high phosphorus diet, increased serum phosphorus levels, as well as cardiac interstitial fibrosis and intramyocardial arterial wall thickness, are higher than in CKD rats fed with a low phosphorus diet [117]. Using the same CKD model fed with high phosphorus diet, increased serum phosphate is present with increased FGF23 (in correlation with increased urinary phosphate excretion) levels and cardiac remodeling determined by increased LV weight and matrix deposition [121]. The degree of cardiac remodeling is correlated with serum phosphate and PTH levels and is attenuated by administration of a phosphate binder [121]. Collectively, these findings suggest that hyperphosphatemia contributes to cardiac changes consistent with uremic cardiomyopathy that could be reversible by dietary phosphate restriction and phosphate binders. Management of CKD-related hyperphosphatemia is practically based on three measures: reducing dietary phosphate intake, use phosphate binders and dialysis treatment. The first two non-invasive measures can start at pre-dialysis stages and could prevent phosphate-induced CV injury. 

### 4.5. Secondary Hyperparathyroidism

PTH, MW 9500 Da, is a peptide hormone which plays a crucial role in systemic calcium homeostasis. Secondary hyperparathyroidism is one of the most problematic manifestations of the CKD-MBD. The severity of secondary hyperparathyroidism increases with decline in renal function [122]. Uremic concentrations of PTH can increase up to 10-fold higher than normal concentrations [21] and removal by conventional hemodialysis is limited. 

In the physiologic state, FGF23 acts on the parathyroid gland by reducing gene expression and secretion. This FGF-23 action is mediated by the activation of the FGF receptor 1c/Klotho receptor complex [123]. However, the mRNA and protein expression of Klotho and FGF receptor 1 in the parathyroid gland decreases with decline in GFR, similar to that in the kidney [71,124]. 

In the absence of Klotho, the parathyroid gland becomes resistant to FGF23, thereby enhancing PTH secretion. On the contrary, PTH secretion is stimulated by decreased 1,25-dihydroxyvitamin D, decreased calcium and increased phosphate levels; all are a consequence of renal resistance to FGF23 due to decreased renal Klotho expression. As previously mentioned, decreased 1,25-dihydroxyvitamin D levels result in parathyroid gland stimulation due to lack of inhibitory signal on PTH gene transcription [70] and possibly, lack of inhibitory feedback to the parathyroid gland by subtle hypocalcemia [61]. Decreased calcium levels enhance PTH gene expression due to an increase in calreticulin, which inhibits the binding of the vitamin D receptor complex to the vitamin D responsive element, thereby preventing the effect of vitamin D on PTH gene transcription. High phosphate levels not only enhance parathyroid secretion [125] but induce parathyroid cell proliferation and parathyroid gland hyperplasia [126] by enhancing cell cycle promotor and progression. The phosphate-induced hyperparathyroidism is alleviated by dietary phosphate restriction, independently of calcium and 1,25-dihydroxyvitamin D levels [127]. In addition, hyperphosphatemia can also inhibit 1-α hydroxylase thereby further decreasing 1,25-dihydroxyvitamin D levels [128]. This suggests that hyperphosphatemia has a big impact on the development of secondary hyperparathyroidism in CKD.

There have been associations between PTH and LV mass [129], CV events [113], CV mortality [130] and all-cause mortality [113] in hemodialysis patients. An association between PTH levels and CV events was also demonstrated in stage 3-4 CKD patients [131]. In addition, a meta-analysis has shown that elevated PTH levels are associated with increased risk for CV events in populations not specific to CKD [132].

Evidence from basic research has shown that PTH contributes to four major cardiovascular effects; contractile disturbance, cardiomyocyte hypertrophy, cardiac interstitial fibrotic and vasodilator effect. The first two cardiac effects are related to increased Ca^2+^ influx, while the vasodilator effect is related to decreased Ca^2+^ influx; such changes in Ca^2+^ influx are controlled by PTH action on L-type Ca^2+^ channels. 

On adult cardiomyocytes, PTH-induced Ca^2+^ influx is mediated by the activation of G_i_ or G_0_ proteins. PTH affects contractile function by inhibiting β-adrenoceptor-mediated contractile effects through activation of the protein kinase C-dependent pathway [133]. Therefore, this effect of PTH is likely to suppress cardiomyocyte contractility. Adult cardiomyocytes stimulated by PTH also show an increase in protein synthesis and re-expression of the fetal type proteins via activation of protein kinase C [133]. This pro-hypertrophic effect of PTH may support the association between LV mass and PTH in ESRD patients with secondary hyperparathyroidism [134]. In addition, LV mass is markedly reduced after parathyroidectomy in patients with secondary parathyroidism with LVH [135]. Cardiac fibrosis is also induced by PTH. In a rat model of combined 5/6 nephrectomy, parathyroidectomy and a high calcium diet, animals with exogenous PTH administration showed higher volume densities of cardiac interstitium and capillaries without a difference in calcium levels, compared with animals without exogenous PTH [136].

## 5. Cardiac Effects of Protein-Bound Molecules

Members in this group are mainly comprised of an indole subgroup such as IS, indole-3-acetic acid (IAA) and phenylacetic acid, a phenol subgroup such as pCS and p-cresyl glucuronide, and others such as homocysteine, hippuratic acid and polyamines. 

Initial skepticism about the toxicity of protein-bound uremic solutes appears to be based upon their non-dialyzable problem together with persistent uremic symptoms in dialysis-dependent CKD patients. There have been evidence indicating that PBUTs are strongly associated with uremia [33] and adverse renal and CV outcomes (Table 1). A causative relationship between PBUTs and adverse biological effects on CV system including their relevant mechanisms has been increasingly demonstrated (Table 1). It is worth noting that adverse clinical outcomes, as well as the biological toxicity of p-cresol, actually represent its conjugates, mainly pCS (>95%) and p-cresyl glucuronide (<4%). Detection of p-cresol is an artifact because its conjugated bonds are hydrolyzed during acid and heat deproteinization in the sample preparation step of measurement [137].

Among PBUTs, IS and pCS/p-cresol have been most extensively investigated with regard to their adverse CV and renal effects. Therefore, this review will discuss PBUTs with a main focus on IS and pCS.

IS and pCS are strongly bound to protein, with the degree of protein-binding of 93% and 95%, respectively [177]. In healthy subjects, the free form of IS is undetectable [21,31] and the concentration approaches zero for free pCS [21]. In uremic patients, both free and total concentrations are substantially increased. Notably, free IS levels remain unchanged when even a small fraction of IS is removed [31,178,179], suggesting a saturation state of IS-albumin binding in uremia.

Clinical studies have shown that increased serum levels of IS and pCS/p-cresol are associated with progression of CKD [155] and CV [138,153,154] and overall mortality [138,153,154,155,163] in both pre-dialysis and dialysis CKD patients. High serum levels of pCS/p-cresol and homocysteine are also predictive of CV events [162,168].

PBUTs are implicated in both atherosclerotic and non-atherosclerotic vascular disease. Pathologic hallmarks of atherosclerosis, including endothelial injury, vascular inflammation and vascular smooth muscle cell (VSMC) proliferation, have been demonstrated to be contributed by PBUTs. Endothelial dysfunction can be induced by IS [143,144], pCS [158] and homocysteine [180]. IS and homocysteine promote vascular inflammatory [174], oxidative stress [144,180] and VSMC proliferation [147,172]. In addition, increased IS levels are associated with vascular stiffness [138], while both IS and pCS levels correlate with vascular calcification in various stages of CKD [138,154]. Experimental studies showed that IS and homocysteine induced osteogenic differentiation of VSMC thereby promoting vascular calcification [148,151,173].

### 5.1. Cardiac Remodeling

Study of the direct cardiac effects of PBUTs has been extremely rare. Phenol was demonstrated several decades ago to suppress contractility of isolated cardiac muscle of piglets [175]. In 2010, direct cardiac effects of IS have been demonstrated for the first time in vitro [140]. Using neonatal rat cardiac myocytes and fibroblasts, treatment with IS increased protein and collagen synthesis determined by ^3^H-leucine and ^3^H-proline incorporation, respectively. IS also upregulated mRNA expression of key cytokines playing role in heart failure progression: IL-1β, IL-6 and TNF-α, in cultured monocytic cells, indicating a pro-inflammatory effect of IS. All of these effects are mediated by the activation of the p38 and p44/42 MAPKs with a downstream mediator nuclear factor-kappa B (NF-κB) [140]. The follow-on study showed that the pro-hypertrophic and pro-fibrotic effects of IS on cardiac cells were inhibited by probenecid and cilastatin [139], organic anion transporters (OATs) 1 and 3 blockers. This suggests that IS is likely implicated in cardiac remodeling by the activation of the p38, p44/42 MAPKs/NF-κB pathway through OATs 1 and 3. In addition, when comparing among IS, pCS and phenylacetic acid, using the same in vitro technique as above [139,140], IS showed the strongest pro-hypertrophic effect, followed by pCS and phenylacetic acid [156]. For the pro-fibrotic effect, IS was more potent than pCS while phenylacetic acid had no effect [156].

Another in vitro study on neonatal rat cardiac myocytes also demonstrated a pro-hypertrophic effect of IS, determined by increased ^3^H-leucine incorporation, cell volume and mRNA expression levels of ANP, BNP and β-MHC, an oxidative stress enhancing effect by increasing mitochondrial ROS production and a decrease in the protein expression of Uncoupling protein 2 (UCP2) and AMP-activated protein kinase (AMPK) [181]. Transfection of UCP2 gene into neonatal rat cardiac myocytes treated with IS diminished cardiomyocyte hypertrophy and oxidative stress. Furthermore, an AMPK activator inhibited IS-induced UCP2 down-regulation and cardiomyocyte hypertrophy. This study suggested that IS induced cardiomyocyte hypertrophy by inhibiting the AMPK/UCP2 pathway.

There have been only few in vivo studies investigating cardiac effects of PBUTs. A study using a 5/6 nephrectomy CKD model demonstrated reduced GFR in CKD rats, as well as increased IS levels, LV diastolic dysfunction, cardiac hypertrophy with increased cardiac mRNA expression of hypertrophic markers (ANP, β-MHC and α-skeletal muscle actin) and cardiac fibrosis with increased cardiac expression of TGF-β, phospho-NF-κB and phospho-p44/42 and phospho-p38 MAPKs [141]. Treatment with an oral sorbent AST-120 reduced serum IS levels, improved renal function and prevented cardiac fibrosis and cardiac protein expression of TGF-β and phospho-NF-κB. In addition, IS levels were correlated with the extent of cardiac fibrosis, independent of blood pressure. This suggests that IS-induced cardiac fibrosis is mediated by the activation of the NF-κB/TGF-β pathway. Another study using a CKD model induced by adriamycin and unilateral nephrectomy showed cardiac fibrosis and hypertrophy, as well as an increase in oxidative stress markers (8-hydroxydeoxyguanosine and acrolein) in cardiac tissue and urine in CKD rats [142]. Treatment with AST-120 significantly reduced cardiac fibrosis and hypertrophy and oxidative stress in association with decreased IS levels. A significant association between cardiac fibrosis and cardiac expression of oxidative stress markers was also demonstrated [142]. This suggests that oxidative stress is implicated in IS-induced cardiac remodeling. 

One study has demonstrated cardiac apoptotic effect of pCS both in vivo and in intro, using a 5/6 nephrectomy CKD mice model and H9c2 cardiac myoblasts, respectively [157]. Administration of pCS showed increased apoptosis and increased interstitial and perivascular fibrosis in non-CKD mice and showed increased apoptosis, a further reduction in LV diastolic function (decreased E/A ratio) and a further increase in interstitial and perivascular fibrosis in CKD mice. The LV diastolic dysfunction induced by pCS in CKD mice was attenuated by inhibiting nicotinamide adenine dinucleotide phosphate (NADPH) oxidase activity. Apoptosis, in association with an upregulation of NADPH oxidase subunit expression and intracellular ROS production, was confirmed in vitro in cultured cardiac myocytes treated with pCS; this apoptosis was also attenuated by inhibiting NADPH oxidase activity [157]. Thus, oxidative stress is implicated in pCS-induced cardiac apoptosis and diastolic dysfunction.

### 5.2. Cardiac Arrhythmias

Recently, the arrhythmogenic effects of IS have been demonstrated in an ex vivo experiment, examining the characteristics of action potentials and intracellular calcium in right and left atrial tissue, pulmonary vein and sinoatrial nodes dissected from rabbits [152]. IS exposure resulted in increased delayed afterdepolarizations, increased burst firings and increased calcium leak in pulmonary vein; decreased spontaneous beating of sinoatrial nodes and shortened action potential durations in left atrium. After stimulation with burst pacing and a β2 agonist isoproterenol, a greater occurrence and a longer duration of AF were observed in left atrial tissue exposed to IS. Such IS-induced arrhythmogenesis was inhibited by an antioxidant, ascorbic acid [152]. This suggests that oxidative stress is involved in IS-induced atrial fibrillation.

Collectively, it is suggested that PBUTs, specifically IS and pCS, are implicated in cardiac remodeling including cardiac fibrosis, cardiac hypertrophy and cardiac apoptosis and arrhythmias. Oxidative stress appears to play a key role in PBUTs-induced cardiac toxicity.

### 5.3. Potential Mechanisms of PBUTs-Induced Cardiotoxicity

Study of direct cardiotoxicity induced by PBUTs is rare compared with renal and vascular toxicity. For the purpose of directing future research on PBUTs-induced cardiotoxicity, it may be assumed that the potential pathways mediating renal and vascular remodeling may also contribute to cardiac remodeling.

Tryptophan metabolites including IS and IAA have been identified as endogenous agonists for the human aryl hydrocarbon receptor (AhR), a ligand-activated transcription factor [182,183]. Recently, the link between vascular effects of IS, specifically pro-oxidative, vascular inflammation and pro-thrombotic effects and AhR activation has been demonstrated. In human umbilical vein endothelial cells, the pro-oxidative effect of IS determined by Nox4 gene expression and superoxide production were inhibited by AhR inhibitors [184]. The inhibition of IS-induced vascular inflammatory effects, including leukocyte recruitment and adhesion and the expression of E-selectin through activator protein-1 transcriptional activity, were found in endothelial cell-specific AhR knockout mice and in vascular endothelial cells transfected with siRNA specific to AhR [185]. Endothelial expression of tissue factor, a coagulative factor, induced by IS and IAA was prevented by an AhR inhibitor or siRNA inhibition [186]. Interestingly, activation of AhR by a solely AhR-mediated exogenous toxin 2,3,7,8-tetrachlorodibenzo-p-dioxin can rapidly induce expression of genes regulating cardiac proliferation, contractility and metabolism [187]. However, whether AhR activation is involved in IS-induced cardiotoxicity is still unknown.

The link between vascular and renal toxicity of IS and Klotho depletion also exists. In endothelial cells stimulated with IS, increased ROS production, reduced nitric oxide (NO) and decreased cell viability are all attenuated by Klotho protein [188]. Renal fibrosis induced by IS and pCS was found in a mild CKD model together with DNA hypermethylation of the Klotho gene and reduced renal Klotho mRNA and protein expression [189]. Reduced Klotho expression by IS and pCS was confirmed by administration of AST-120, an IS and pCS lowering agent [190]. ROS and NF-κB have been demonstrated to be involved in the mechanism of IS-reduced Klotho expression in the kidney [191]. This suggests that Klotho deficiency might be involved in PBUT-induced renal fibrosis mediated via the ROS/NF-κB pathway. Interestingly, a decrease in cardiac Klotho expression in correlation with an increase in cardiac fibrosis and cardiac expression of NF-κB, inducible nitric oxide synthase and TGF-β1 has been recently demonstrated in patients at high risk of CVD [192]. However, the link between IS-induced cardiotoxicity and cardiac Klotho status has never been investigated.

### 5.4. Targeting PBUTs

Targeting PBUTs can be achieved by 2 strategies: prevention of toxin production and toxin removal by renal replacement therapy. Increased circulating levels of PBUTs can occur at early stage 3 CKD [193] and PBUTs are non-dialyzable. Thus, targeting toxin production at early stage CKD appears to be greatly beneficial in terms of preventing the development and progression of CV pathology, especially fibrosis-related organ damage that may be irreversible.

Uremic retention solutes may be alternatively classified according to their origin into 3 groups: endogenous metabolism, microbial metabolism and exogenous intake [194]. Many PBUTs including IS and pCS are derived from colonic microbial metabolism. Therefore, targeting colon can prevent the production of colon-derived PBUTs. This can be achieved by several measures.

***Low-protein diet*** is to decrease amino acid substrates for PBUT production. Specifically, tryptophan is a substrate for phenol subgroup while tyrosine and phenylalanine are for phenol subgroup. These amino acids are commonly found in basic foods. Nevertheless, a reduction in IS production has been demonstrated in pre-dialysis CKD patients with a very low protein diet [195].

***Maintenance of normal gut homeostasis by probiotics, prebiotics, synbiotic or laxatives.*** A shift of colonic microbiome, impaired intestinal absorption and prolonged colon transit time in the setting of CKD results in an increase in amino acid substrates for colonic bacteria to produce the precursors (indoles and phenols) of PBUTs. Probiotics [196,197] using good live microorganisms, prebiotics [198] using non-digestible dietary fiber to modify microbial environment, synbiotics [199,200] using combined probiotic and prebiotic treatment and laxatives [201] for accelerating colon transit of amino acid substrates, have been all demonstrated to reduce circulating levels of PBUTs including IS, pCS and p-cresol in both CKD patients and animal models.

***Oral sorbents*** adsorb PBUT precursors which are synthesized from amino acid substrates by colonic bacteria. AST-120 is the most used sorbent to reduce colon-derived PBUTs due to its high adsorptive capacity to low molecular weight molecules without interfering with intestinal enzymes and nutrition [202,203]. AST-120 has been demonstrated to reduce PBUTs levels in association with improved renal function and renal pathologic changes [203,204,205], delay progression of atherosclerosis [206] and improved cardiac remodeling [141,142].

However, the beneficial of AST-120 is still controversial. In a renal failure mouse model induced by adenine, AST-120 did not show an improvement in renal function and fibrosis despite a significant reduction in IS and pCS accumulation in the renal tissue [207]. Large scale randomized controlled trials in moderate to severe CKD (pre-dialysis) patients failed to show renal benefits of AST-120 on the delay in CKD progression despite a significantly better preservation of renal function in AST-120 treated than placebo groups [208,209]. The first phase III multicenter, randomized placebo-controlled trial (*n* = 460) showed comparable low numbers of primary end-point events (serum creatinine doubling or increasing to 6 mg/dL, need for renal replacement therapy and death) within the follow-up time of only one year [208]. Another study consisted of two randomized placebo-controlled EPPIC-1 and EPPIC-2 trials (*n* = 2035) using the composite endpoint of dialysis initiation, kidney transplantation and serum creatinine doubling had similar limitations on infrequent primary endpoint events due to a substantially longer actual time to primary endpoint events than estimated (189 vs 135.6 weeks for EPPIC-1 and 170.3 vs 150 weeks for EPPIC-2) [209]. This indicates a failure to select patients with progressive CKD. The authors suggested to selectively recruit patients with a urine protein/urine creatinine ratio of ≥1.0 and positive hematuria who are at high risk of CKD progression for the future studies [209]. Thus, study with adjusted inclusion criteria and probably longer follow-up, is needed to further clarify the benefit of AST-120 in CKD patients. It is interesting to also include CV endpoints.

## 6. Conclusions

Accumulation of uremic toxins is a hallmark of CKD progression. As CKD is a progressive disorder by nature, uremic toxin retention is worsening over time without interventions. A growing body of evidence indicates that uremic toxins are responsible for CVD, a major cause of death in the CKD population. The coexistence of CKD and CV complications, recognized as type 4 cardiorenal syndrome, further accelerates failure of both organs, leading to a multiplicative increase risk of death. Given that uremic toxins with putative cardiovascular toxicity including FGF23 and PBUTs starts to accumulate in the body since early stage CKD, elimination no longer relies on only renal replacement therapy. Targeting such toxins and/or their relevant mediators at pre-dialysis stages may help breaking the vicious cycle of the cardio-renal connection. In this regard, tremendous work needs to be done as the (off-target) biological activity on the heart has not yet or not fully investigated in a number of uremic toxins.

## Figures and Tables

**Table 1:** Adverse cardiovascular outcomes and biological effects of protein bound uremic toxins (PBUTs).

Compounds	Cardiovascular Outcomes	Cardiac Effects	Vascular Effects
**Indoxyl Sulfate**	- Increase risk of cardiovascular and all-cause mortality [138]	**In vitro** - Pro-fibrotic and pro-hypertrophic effects in NCF and NCM, respectively, with OATs 1 and 3 as potential intracellular transporters of IS [139] - Pro-fibrotic, pro-hypertrophic and pro-inflammatory effects are mediated via the p38 & p44/42 MAPKs/NF-κB pathway [140] **In vivo** - Cardiac fibrosis in association with diastolic dysfunction via TGF-β/ NF-κB pathway in a 5/6 nephrectomy model [141] - Cardiac fibrosis and hypertrophy in association with increased cardiac oxidative stress in 1/2 nephrectomy plus adriamycin and 5/6 nephrectomy models [142]	**In vitro** - Defective endothelial proliferation and wound repair without effects on cell viability and apoptosis in cultured HUVECs [143] - Enhance oxidative stress [144,145], inhibit cell proliferation [145] and promote cell senescence [145] in cultured HUVECs; all attenuated by OAT inhibitor probenecid and antioxidants [145] - Promote cell senescence in cultured HUVECs via AhR [146] - Promote rat VSMC proliferation mediated via OAT3-p44/42 MAPK pathway and increase PDGF-C and PDGF-β receptor gene expression [147] - Promote ROS generation and osteoblastic transformation mediated by NADPH oxidase activation in human aortic smooth muscle cells [148] - Induce IL-6 protein expression via OAT3/AhR/NF-κB pathway in human vascular endothelial and smooth muscle cells [149] **In vivo** - Induce IL-6 protein expression in aortic tissue of IS-administered rats [149] - Enhance endothelial-leukocyte adhesion, leukocyte extravasation and interrupted blood flow without a vasoactive effect, fibrin deposition or capillary plugging in rats with IS-peritoneal superfusion [150] - Promote aortic calcification and wall thickness in association with cell senescence in IS-administered hypertensive rats [151] **Ex vivo** - Induce atrial fibrillation in dissected atrial tissue [152]
**p-cresyl Sulfate**	- Increase risk of cardiovascular [153,154] and all-cause mortality [154,155]	**In vitro** - Pro-fibrotic and pro-hypertrophic effects [156] **In vivo** - Diastolic dysfunction in association with cardiac fibrosis, apoptosis and oxidative stress in pCS-administered uremic rats [157]	**In vitro** - Promote endothelial dysfunction by inducing Rho kinase-mediated microparticle shedding in HUVECs [158] - Increase endothelial permeability to albumin in the presence of p-cresyl glucuronide [159] - Increase leukocytes oxidative burst activity in healthy human blood exposed to pCS [160,161] **In vivo** - Impair blood flow with vascular leakage, in the presence of p-cresyl glucuronide in peritoneal toxin-superfusion model [150]
**p-cresol** (present in the body as its conjugated forms, mainly p-cresyl sulfate)	- Increase risk of cardiovascular events [162] and all-cause mortality [163]	**In vitro** - Pro-hypertrophic effects in cultured cardiac myocytes [156] - Reduce spontaneous contraction with enhanced irregular beating of cardiac myocytes in association with abnormal structural and functional changes in the gap junction [164] **In vivo** - n/a	**In vitro** - n/a - Defective endothelial proliferation and wound repair without effects on cell viability and apoptosis in cultured HUVECs [143] - Defective leukocyte adhesion and inhibit endothelial adhesion molecule expression in cytokine-stimulated HUVECs [165] **In vivo** - n/a
**Phenylacetic Acid**	- n/a	- Increased protein synthesis in NCM in vitro [140]	- n/a
**Indole-3-acetic Acid**	- Increase risk of cardiovascular events and all-cause mortality [166]	- n/a	**In vitro** - Induce CD133+ cell apoptosis in vitro [167] - Induce expression of cyclooxygenase-2 pro–inflammatory enzyme in HUVECs via AhR/p38MAPK/NF-κB pathway [166]
**Homocysteine**	- Increase risk of CV events [168,169,170], not prevented by folic acid based regimens [171] and all-cause mortality [168,170]	- n/a	**In vitro** - MAPK-mediated VSMC proliferation [172] - Promote calcium deposition and osteogenic differentiation in VSMCs co–cultured with THP-1 cells (human leukemia monocytic cell line) [173] **In vivo** - Increased expression of vascular inflammatory and thrombogenic mediators [174]
**Hippuric Acid**	- n/a	- n/a	- n/a
**Phenol**	- n/a	**In vitro** - suppress contractility of cardiac muscle [175]	- n/a
**Hydroquinone**	- n/a	- n/a	**In vitro** - Non-apoptotic cell death in cultured human microvascular endothelial cells [176]

## References

[1] Srivastava A., Kaze A.D., McMullan C.J., Isakova T., Waikar S.S. (2017). Uric acid and the risks of kidney failure and death in individuals with ckd. Am. J. Kidney Dis..

[2] Schiffrin E.L., Lipman M.L., Mann J.F. (2007). Chronic kidney disease: Effects on the cardiovascular system. Circulation.

[3] Levey A.S., Eckardt K.U., Tsukamoto Y., Levin A., Coresh J., Rossert J., De Zeeuw D., Hostetter T.H., Lameire N., Eknoyan G. (2005). Definition and classification of chronic kidney disease: A position statement from kidney disease: Improving global outcomes (kdigo). Kidney Int..

[4] Keith D.S., Nichols G.A., Gullion C.M., Brown J.B., Smith D.H. (2004). Longitudinal follow-up and outcomes among a population with chronic kidney disease in a large managed care organization. Arch. Intern Med..

[5] Schrier R.W. (2007). Cardiorenal versus renocardiac syndrome: Is there a difference?. Nat. Clin. Pract. Nephrol..

[6] U.S. Renal Data System (2007). USRDS 2007 Annual Data Report: Atlas of Chronic Kidney Disease and End-Stage Renal Disease in the United States.

[7] Sarnak M.J., Levey A.S., Schoolwerth A.C., Coresh J., Culleton B., Hamm L.L., McCullough P.A., Kasiske B.L., Kelepouris E., Klag M.J. (2003). Kidney disease as a risk factor for development of cardiovascular disease: A statement from the american heart association councils on kidney in cardiovascular disease, high blood pressure research, clinical cardiology and epidemiology and prevention. Circulation.

[8] U.S. Renal Data System (2012). USRDS 2012 Annual Data Report: Atlas of Chronic Kidney Disease and End-Stage Renal Disease in the United States.

[9] Foley R.N., Parfrey P.S., Harnett J.D., Kent G.M., Martin C.J., Murray D.C., Barre P.E. (1995). Clinical and echocardiographic disease in patients starting end-stage renal disease therapy. Kidney Int..

[10] Roberts P.R., Green D. (2011). Arrhythmias in chronic kidney disease. Heart.

[11] Pun P.H., Smarz T.R., Honeycutt E.F., Shaw L.K., Al-Khatib S.M., Middleton J.P. (2009). Chronic kidney disease is associated with increased risk of sudden cardiac death among patients with coronary artery disease. Kidney Int..

[12] Reinecke H., Brand E., Mesters R., Schabitz W.R., Fisher M., Pavenstadt H., Breithardt G. (2009). Dilemmas in the management of atrial fibrillation in chronic kidney disease. J. Am. Soc. Nephrol..

[13] Lindner A., Charra B., Sherrard D.J., Scribner B.H. (1974). Accelerated atherosclerosis in prolonged maintenance hemodialysis. N. Eng. J. Med..

[14] Rostand S.G., Kirk K.A., Rutsky E.A. (1984). Dialysis-associated ischemic heart disease: Insights from coronary angiography. Kidney Int..

[15] Glassock R.J., Pecoits-Filho R., Barberato S.H. (2009). Left ventricular mass in chronic kidney disease and esrd. Clin. J. Am. Soc. Nephrol..

[16] Amann K., Neususs R., Ritz E., Irzyniec T., Wiest G., Mall G. (1995). Changes of vascular architecture independent of blood pressure in experimental uremia. Am. J. Hypertens..

[17] Guerin A.P., Pannier B., Marchais S.J., London G.M. (2006). Cardiovascular disease in the dialysis population: Prognostic significance of arterial disorders. Curr. Opin. Nephrol. Hypertens..

[18] U.S. Renal Data System (2010). USRDS 2010 Annual Data Report: Atlas of Chronic Kidney Disease and End-Stage Renal Disease in the United States.

[19] Vanholder R., De Smet R., Glorieux G., Argiles A., Baurmeister U., Brunet P., Clark W., Cohen G., De Deyn P.P., Deppisch R. (2003). Review on uremic toxins: Classification, concentration and interindividual variability. Kidney Int..

[20] Meert N., Schepers E., De Smet R., Argiles A., Cohen G., Deppisch R., Drueke T., Massy Z., Spasovski G., Stegmayr B. (2007). Inconsistency of reported uremic toxin concentrations. Artif. Organs.

[21] Duranton F., Cohen G., De Smet R., Rodriguez M., Jankowski J., Vanholder R., Argiles A. (2012). Normal and pathologic concentrations of uremic toxins. J. Am. Soc. Nephrol..

[22] Zoccali C., Benedetto F.A., Mallamaci F., Tripepi G., Giacone G., Stancanelli B., Cataliotti A., Malatino L.S. (2004). Left ventricular mass monitoring in the follow-up of dialysis patients: Prognostic value of left ventricular hypertrophy progression. Kidney Int..

[23] London G.M., Pannier B., Guerin A.P., Blacher J., Marchais S.J., Darne B., Metivier F., Adda H., Safar M.E. (2001). Alterations of left ventricular hypertrophy in and survival of patients receiving hemodialysis: Follow-up of an interventional study. J. Am. Soc. Nephrol..

[24] Gross M.L., Ritz E. (2008). Hypertrophy and fibrosis in the cardiomyopathy of uremia—Beyond coronary heart disease. Semin. Dial..

[25] Wali R.K., Wang G.S., Gottlieb S.S., Bellumkonda L., Hansalia R., Ramos E., Drachenberg C., Papadimitriou J., Brisco M.A., Blahut S. (2005). Effect of kidney transplantation on left ventricular systolic dysfunction and congestive heart failure in patients with end-stage renal disease. J. Am. Coll. Cardiol..

[26] Dhondt A., Vanholder R., Van Biesen W., Lameire N. (2000). The removal of uremic toxins. Kidney Int. Suppl..

[27] Makita Z., Bucala R., Rayfield E.J., Friedman E.A., Kaufman A.M., Korbet S.M., Barth R.H., Winston J.A., Fuh H., Manogue K.R. (1994). Reactive glycosylation endproducts in diabetic uraemia and treatment of renal failure. Lancet.

[28] Meert N., Eloot S., Waterloos M.A., Van Landschoot M., Dhondt A., Glorieux G., Ledebo I., Vanholder R. (2009). Effective removal of protein-bound uraemic solutes by different convective strategies: A prospective trial. Nephrol. Dial. Transpl..

[29] Ikegaya K., Nokihara K., Yasuhara T. (2010). Characterization of sulfhydryl heterogeneity in human serum albumin and recombinant human serum albumin for clinical use. Biosci. Biotechnol. Biochem..

[30] Fehske K.J., Muller W.E., Platt K.L., Stillbauer A.E. (1981). Inhibition of benzodiazepine receptor binding by several tryptophan ad indole derivatives. Biochem. Pharmacol..

[31] Niwa T., Takeda N., Tatematsu A., Maeda K. (1988). Accumulation of indoxyl sulfate, an inhibitor of drug-binding, in uremic serum as demonstrated by internal-surface reversed-phase liquid chromatography. Clin. Chem..

[32] Sarnatskaya V.V., Yushko L.A., Sakhno L.A., Nikolaev V.G., Nikolaev A.V., Grinenko D.V., Mikhalovsky S.V. (2007). New approaches to the removal of protein-bound toxins from blood plasma of uremic patients. Artif. Cells Blood Substit. Immobil. Biotechnol..

[33] Bammens B., Evenepoel P., Verbeke K., Vanrenterghem Y. (2003). Removal of middle molecules and protein-bound solutes by peritoneal dialysis and relation with uremic symptoms. Kidney Int..

[34] Weisensee D., Low-Friedrich I., Riehle M., Bereiter-Hahn J., Schoeppe W. (1993). In vitro approach to ‘uremic cardiomyopathy‘. Nephron.

[35] Kersting F., Brass H., Heintz R. (1978). Uremic cardiomyopathy: Studies on cardiac function in the guinea pig. Clin. Nephrol..

[36] Vanholder R., Gryp T., Glorieux G. (2018). Urea and chronic kidney disease: The comeback of the century? (in uraemia research). Nephrol. Dial. Transpl..

[37] Wang Z., Nicholls S.J., Rodriguez E.R., Kummu O., Horkko S., Barnard J., Reynolds W.F., Topol E.J., DiDonato J.A., Hazen S.L. (2007). Protein carbamylation links inflammation, smoking, uremia and atherogenesis. Nat. Med..

[38] Drechsler C., Kalim S., Wenger J.B., Suntharalingam P., Hod T., Thadhani R.I., Karumanchi S.A., Wanner C., Berg A.H. (2015). Protein carbamylation is associated with heart failure and mortality in diabetic patients with end-stage renal disease. Kidney Int..

[39] Schlesinger S., Sonntag S.R., Lieb W., Maas R. (2016). Asymmetric and symmetric dimethylarginine as risk markers for total mortality and cardiovascular outcomes: A systematic review and meta-analysis of prospective studies. PLoS ONE.

[40] Zoccali C., Bode-Boger S., Mallamaci F., Benedetto F., Tripepi G., Malatino L., Cataliotti A., Bellanuova I., Fermo I., Frolich J. (2001). Plasma concentration of asymmetrical dimethylarginine and mortality in patients with end-stage renal disease: A prospective study. Lancet.

[41] Liu X., Xu X., Shang R., Chen Y. (2018). Asymmetric dimethylarginine (adma) as an important risk factor for the increased cardiovascular diseases and heart failure in chronic kidney disease. Nitric Oxide.

[42] Wright A.F., Rudan I., Hastie N.D., Campbell H. (2010). A ‘complexity‘ of urate transporters. Kidney Int..

[43] Dong J., Han Q.F., Zhu T.Y., Ren Y.P., Chen J.H., Zhao H.P., Chen M.H., Xu R., Wang Y., Hao C.M. (2014). The associations of uric acid, cardiovascular and all-cause mortality in peritoneal dialysis patients. PLoS ONE.

[44] Forman J.P., Choi H., Curhan G.C. (2009). Uric acid and insulin sensitivity and risk of incident hypertension. Arch. Intern Med..

[45] Ekundayo O.J., Dell‘Italia L.J., Sanders P.W., Arnett D., Aban I., Love T.E., Filippatos G., Anker S.D., Lloyd-Jones D.M., Bakris G. (2010). Association between hyperuricemia and incident heart failure among older adults: A propensity-matched study. Int. J. Cardiol..

[46] Tamariz L., Hernandez F., Bush A., Palacio A., Hare J.M. (2014). Association between serum uric acid and atrial fibrillation: A systematic review and meta-analysis. Heart Rhythm.

[47] Chaudhary K., Malhotra K., Sowers J., Aroor A. (2013). Uric acid—Key ingredient in the recipe for cardiorenal metabolic syndrome. Cardiorenal Med..

[48] El Ridi R., Tallima H. (2017). Physiological functions and pathogenic potential of uric acid: A review. J. Adv. Res..

[49] Tang W.H., Wang Z., Levison B.S., Koeth R.A., Britt E.B., Fu X., Wu Y., Hazen S.L. (2013). Intestinal microbial metabolism of phosphatidylcholine and cardiovascular risk. N. Eng. J. Med..

[50] Wang Z., Klipfell E., Bennett B.J., Koeth R., Levison B.S., Dugar B., Feldstein A.E., Britt E.B., Fu X., Chung Y.M. (2011). Gut flora metabolism of phosphatidylcholine promotes cardiovascular disease. Nature.

[51] Qi J., You T., Li J., Pan T., Xiang L., Han Y., Zhu L. (2018). Circulating trimethylamine n-oxide and the risk of cardiovascular diseases: A systematic review and meta-analysis of 11 prospective cohort studies. J. Cell Mol. Med..

[52] Schiattarella G.G., Sannino A., Toscano E., Giugliano G., Gargiulo G., Franzone A., Trimarco B., Esposito G., Perrino C. (2017). Gut microbe-generated metabolite trimethylamine-n-oxide as cardiovascular risk biomarker: A systematic review and dose-response meta-analysis. Eur. Heart. J..

[53] Tang W.H., Wang Z., Kennedy D.J., Wu Y., Buffa J.A., Agatisa-Boyle B., Li X.S., Levison B.S., Hazen S.L. (2015). Gut microbiota-dependent trimethylamine n-oxide (tmao) pathway contributes to both development of renal insufficiency and mortality risk in chronic kidney disease. Circ. Res..

[54] Manghat P., Sodi R., Swaminathan R. (2014). Phosphate homeostasis and disorders. Ann. Clin. Biochem..

[55] Moe S., Drueke T., Cunningham J., Goodman W., Martin K., Olgaard K., Ott S., Sprague S., Lameire N., Eknoyan G. (2006). Definition, evaluation and classification of renal osteodystrophy: A position statement from kidney disease: Improving global outcomes (kdigo). Kidney Int..

[56] Gutierrez O.M., Mannstadt M., Isakova T., Rauh-Hain J.A., Tamez H., Shah A., Smith K., Lee H., Thadhani R., Juppner H. (2008). Fibroblast growth factor 23 and mortality among patients undergoing hemodialysis. N. Eng. J. Med..

[57] Fenton S.S., Schaubel D.E., Desmeules M., Morrison H.I., Mao Y., Copleston P., Jeffery J.R., Kjellstrand C.M. (1997). Hemodialysis versus peritoneal dialysis: A comparison of adjusted mortality rates. Am. J. Kidney Dis..

[58] Maggi E., Bellazzi R., Falaschi F., Frattoni A., Perani G., Finardi G., Gazo A., Nai M., Romanini D., Bellomo G. (1994). Enhanced ldl oxidation in uremic patients: An additional mechanism for accelerated atherosclerosis?. Kidney Int..

[59] Klinkner A.M., Waites C.R., Kerns W.D., Bugelski P.J. (1995). Evidence of foam cell and cholesterol crystal formation in macrophages incubated with oxidized ldl by fluorescence and electron microscopy. J. Histochem. Cytochem..

[60] Vanholder R., Argiles A., Baurmeister U., Brunet P., Clark W., Cohen G., De Deyn P.P., Deppisch R., Descamps-Latscha B., Henle T. (2001). Uremic toxicity: Present state of the art. Int. J. Artif. Organs..

[61] Wolf M. (2012). Update on fibroblast growth factor 23 in chronic kidney disease. Kidney Int..

[62] Hasegawa H., Nagano N., Urakawa I., Yamazaki Y., Iijima K., Fujita T., Yamashita T., Fukumoto S., Shimada T. (2010). Direct evidence for a causative role of fgf23 in the abnormal renal phosphate handling and vitamin d metabolism in rats with early-stage chronic kidney disease. Kidney Int..

[63] Ben-Dov I.Z., Galitzer H., Lavi-Moshayoff V., Goetz R., Kuro-o M., Mohammadi M., Sirkis R., Naveh-Many T., Silver J. (2007). The parathyroid is a target organ for fgf23 in rats. J. Clin. Investig..

[64] Verkaik M., Oranje M., Abdurrachim D., Goebel M., Gam Z., Prompers J.J., Helmes M., Ter Wee P.M., van der Velden J., Kuster D.W. (2018). High fibroblast growth factor 23 concentrations in experimental renal failure impair calcium handling in cardiomyocytes. Physiol. Rep..

[65] Faul C., Amaral A.P., Oskouei B., Hu M.C., Sloan A., Isakova T., Gutierrez O.M., Aguillon-Prada R., Lincoln J., Hare J.M. (2011). Fgf23 induces left ventricular hypertrophy. J. Clin. Investig..

[66] Isakova T., Wahl P., Vargas G.S., Gutierrez O.M., Scialla J., Xie H., Appleby D., Nessel L., Bellovich K., Chen J. (2011). Fibroblast growth factor 23 is elevated before parathyroid hormone and phosphate in chronic kidney disease. Kidney Int..

[67] Zhang L.N., Yang G., Cheng C., Shen C., Cui Y.Y., Zhang J., Zhang J.J., Shen Z.X., Zeng M., Ge Y.F. (2015). Plasma fgf23 levels and heart rate variability in patients with stage 5 ckd. Osteoporos. Int..

[68] Christov M., Waikar S.S., Pereira R.C., Havasi A., Leaf D.E., Goltzman D., Pajevic P.D., Wolf M., Juppner H. (2013). Plasma fgf23 levels increase rapidly after acute kidney injury. Kidney Int..

[69] Mace M.L., Gravesen E., Nordholm A., Hofman-Bang J., Secher T., Olgaard K., Lewin E. (2017). Kidney fibroblast growth factor 23 does not contribute to elevation of its circulating levels in uremia. Kidney Int..

[70] Silver J., Russell J., Sherwood L.M. (1985). Regulation by vitamin d metabolites of messenger ribonucleic acid for preproparathyroid hormone in isolated bovine parathyroid cells. Proc. Natl. Acad. Sci. USA.

[71] Krajisnik T., Olauson H., Mirza M.A., Hellman P., Akerstrom G., Westin G., Larsson T.E., Bjorklund P. (2010). Parathyroid klotho and fgf-receptor 1 expression decline with renal function in hyperparathyroid patients with chronic kidney disease and kidney transplant recipients. Kidney Int..

[72] Sakan H., Nakatani K., Asai O., Imura A., Tanaka T., Yoshimoto S., Iwamoto N., Kurumatani N., Iwano M., Nabeshima Y. (2014). Reduced renal alpha-klotho expression in ckd patients and its effect on renal phosphate handling and vitamin d metabolism. PLoS ONE.

[73] Kuro-o M. (2012). Klotho in health and disease. Curr. Opin. Nephrol. Hypertens..

[74] Richter B., Faul C. (2018). Fgf23 actions on target tissues-with and without klotho. Front. Endocrinol..

[75] Hsu H.J., Wu M.S. (2009). Fibroblast growth factor 23: A possible cause of left ventricular hypertrophy in hemodialysis patients. Am. J. Med. Sci..

[76] Jovanovich A., Ix J.H., Gottdiener J., McFann K., Katz R., Kestenbaum B., de Boer I.H., Sarnak M., Shlipak M.G., Mukamal K.J. (2013). Fibroblast growth factor 23, left ventricular mass and left ventricular hypertrophy in community-dwelling older adults. Atherosclerosis.

[77] Gutierrez O.M., Januzzi J.L., Isakova T., Laliberte K., Smith K., Collerone G., Sarwar A., Hoffmann U., Coglianese E., Christenson R. (2009). Fibroblast growth factor 23 and left ventricular hypertrophy in chronic kidney disease. Circulation.

[78] Kim H.J., Park M., Park H.C., Jeong J.C., Kim D.K., Joo K.W., Hwang Y.H., Yang J., Ahn C., Oh K.H. (2016). Baseline fgf23 is associated with cardiovascular outcome in incident pd patients. Perit. Dial. Int..

[79] Marthi A., Donovan K., Haynes R., Wheeler D.C., Baigent C., Rooney C.M., Landray M.J., Moe S.M., Yang J., Holland L. (2018). Fibroblast growth factor-23 and risks of cardiovascular and noncardiovascular diseases: A meta-analysis. J. Am. Soc. Nephrol..

[80] Seiler S., Rogacev K.S., Roth H.J., Shafein P., Emrich I., Neuhaus S., Floege J., Fliser D., Heine G.H. (2014). Associations of fgf-23 and sklotho with cardiovascular outcomes among patients with ckd stages 2-4. Clin. J. Am. Soc. Nephrol..

[81] Nakano C., Hamano T., Fujii N., Obi Y., Matsui I., Tomida K., Mikami S., Inoue K., Shimomura A., Nagasawa Y. (2012). Intact fibroblast growth factor 23 levels predict incident cardiovascular event before but not after the start of dialysis. Bone.

[82] Fliser D., Kollerits B., Neyer U., Ankerst D.P., Lhotta K., Lingenhel A., Ritz E., Kronenberg F., Kuen E., Konig P. (2007). Fibroblast growth factor 23 (fgf23) predicts progression of chronic kidney disease: The mild to moderate kidney disease (mmkd) study. J. Am. Soc. Nephrol..

[83] Panwar B., Judd S.E., Wadley V.G., Jenny N.S., Howard V.J., Safford M.M., Gutierrez O.M. (2018). Association of fibroblast growth factor 23 with risk of incident coronary heart disease in community-living adults. JAMA Cardiol..

[84] Hao H., Li X., Li Q., Lin H., Chen Z., Xie J., Xuan W., Liao W., Bin J., Huang X. (2016). Fgf23 promotes myocardial fibrosis in mice through activation of beta-catenin. Oncotarget.

[85] Andrukhova O., Slavic S., Odorfer K.I., Erben R.G. (2015). Experimental myocardial infarction upregulates circulating fibroblast growth factor-23. J. Bone Miner. Res..

[86] Andersen I.A., Huntley B.K., Sandberg S.S., Heublein D.M., Burnett J.C. (2016). Elevation of circulating but not myocardial fgf23 in human acute decompensated heart failure. Nephrol. Dial. Transpl..

[87] Ter Maaten J.M., Voors A.A., Damman K., van der Meer P., Anker S.D., Cleland J.G., Dickstein K., Filippatos G., van der Harst P., Hillege H.L. (2018). Fibroblast growth factor 23 is related to profiles indicating volume overload, poor therapy optimization and prognosis in patients with new-onset and worsening heart failure. Int. J. Cardiol..

[88] Di Giuseppe R., Kuhn T., Hirche F., Buijsse B., Dierkes J., Fritsche A., Kaaks R., Boeing H., Stangl G.I., Weikert C. (2015). Plasma fibroblast growth factor 23 and risk of cardiovascular disease: Results from the epic-germany case-cohort study. Eur. J. Epidemiol..

[89] Imazu M., Takahama H., Asanuma H., Funada A., Sugano Y., Ohara T., Hasegawa T., Asakura M., Kanzaki H., Anzai T. (2014). Pathophysiological impact of serum fibroblast growth factor 23 in patients with nonischemic cardiac disease and early chronic kidney disease. Am. J. Physiol. Heart Circ. Physiol..

[90] Touchberry C.D., Green T.M., Tchikrizov V., Mannix J.E., Mao T.F., Carney B.W., Girgis M., Vincent R.J., Wetmore L.A., Dawn B. (2013). Fgf23 is a novel regulator of intracellular calcium and cardiac contractility in addition to cardiac hypertrophy. Am. J. Physiol. Endocrinol. Metab..

[91] Huang S.Y., Chen Y.C., Kao Y.H., Hsieh M.H., Lin Y.K., Chung C.C., Lee T.I., Tsai W.C., Chen S.A., Chen Y.J. (2016). Fibroblast growth factor 23 dysregulates late sodium current and calcium homeostasis with enhanced arrhythmogenesis in pulmonary vein cardiomyocytes. Oncotarget.

[92] Grabner A., Amaral A.P., Schramm K., Singh S., Sloan A., Yanucil C., Li J., Shehadeh L.A., Hare J.M., David V. (2015). Activation of cardiac fibroblast growth factor receptor 4 causes left ventricular hypertrophy. Cell Metab..

[93] Grabner A., Schramm K., Silswal N., Hendrix M., Yanucil C., Czaya B., Singh S., Wolf M., Hermann S., Stypmann J. (2017). Fgf23/fgfr4-mediated left ventricular hypertrophy is reversible. Sci. Rep..

[94] Shalhoub V., Shatzen E.M., Ward S.C., Davis J., Stevens J., Bi V., Renshaw L., Hawkins N., Wang W., Chen C. (2012). Fgf23 neutralization improves chronic kidney disease-associated hyperparathyroidism yet increases mortality. J. Clin. Investig..

[95] Di Marco G.S., Reuter S., Kentrup D., Grabner A., Amaral A.P., Fobker M., Stypmann J., Pavenstadt H., Wolf M., Faul C. (2014). Treatment of established left ventricular hypertrophy with fibroblast growth factor receptor blockade in an animal model of CKD. Nephrol. Dial. Transplant..

[96] Carlson N., Mortensen O.H., Axelsen M., Pedersen R.S., Heaf J.G. (2017). Clearance of sclerostin, osteocalcin, fibroblast growth factor 23 and osteoprotegerin by dialysis. Blood Purif..

[97] Uhlin F., Magnusson P., Larsson T.E., Fernstrom A. (2015). In the backwater of convective dialysis: Decreased 25-hydroxyvitamin d levels following the switch to online hemodiafiltration. Clin. Nephrol..

[98] Smith E.R., Holt S.G., Hewitson T.D. (2017). Fgf23 activates injury-primed renal fibroblasts via fgfr4-dependent signalling and enhancement of tgf-beta autoinduction. Int. J. Biochem. Cell Biol..

[99] Qiao X., Rao P., Zhang Y., Liu L., Pang M., Wang H., Hu M., Tian X., Zhang J., Zhao Y. (2017). Redirecting tgf-beta signaling through the beta-catenin/foxo complex prevents kidney fibrosis. J. Am. Soc. Nephrol..

[100] Seiler S., Cremers B., Rebling N.M., Hornof F., Jeken J., Kersting S., Steimle C., Ege P., Fehrenz M., Rogacev K.S. (2011). The phosphatonin fibroblast growth factor 23 links calcium-phosphate metabolism with left-ventricular dysfunction and atrial fibrillation. Eur. Heart. J..

[101] Mehta R., Cai X., Lee J., Scialla J.J., Bansal N., Sondheimer J.H., Chen J., Hamm L.L., Ricardo A.C., Navaneethan S.D. (2016). Association of fibroblast growth factor 23 with atrial fibrillation in chronic kidney disease, from the chronic renal insufficiency cohort study. JAMA Cardiol..

[102] Mathew J.S., Sachs M.C., Katz R., Patton K.K., Heckbert S.R., Hoofnagle A.N., Alonso A., Chonchol M., Deo R., Ix J.H. (2014). Fibroblast growth factor-23 and incident atrial fibrillation: The multi-ethnic study of atherosclerosis (mesa) and the cardiovascular health study (chs). Circulation.

[103] Wu Y., Wang L., Ma J., Song Y., Zhang P., Luo A., Fu C., Cao Z., Wang X., Shryock J.C. (2015). Protein kinase C and Ca(2+) -calmodulin-dependent protein kinase ii mediate the enlarged reverse incx induced by ouabain-increased late sodium current in rabbit ventricular myocytes. Exp. Physiol..

[104] Rossaint J., Oehmichen J., Van Aken H., Reuter S., Pavenstadt H.J., Meersch M., Unruh M., Zarbock A. (2016). Fgf23 signaling impairs neutrophil recruitment and host defense during ckd. J. Clin. Investig..

[105] Leifheit-Nestler M., Grabner A., Hermann L., Richter B., Schmitz K., Fischer D.C., Yanucil C., Faul C., Haffner D. (2017). Vitamin d treatment attenuates cardiac fgf23/fgfr4 signaling and hypertrophy in uremic rats. Nephrol. Dial. Transpl..

[106] Chen S., Law C.S., Grigsby C.L., Olsen K., Hong T.T., Zhang Y., Yeghiazarians Y., Gardner D.G. (2011). Cardiomyocyte-specific deletion of the vitamin d receptor gene results in cardiac hypertrophy. Circulation.

[107] Bodyak N., Ayus J.C., Achinger S., Shivalingappa V., Ke Q., Chen Y.S., Rigor D.L., Stillman I., Tamez H., Kroeger P.E. (2007). Activated vitamin d attenuates left ventricular abnormalities induced by dietary sodium in dahl salt-sensitive animals. Proc. Natl. Acad. Sci. USA.

[108] Kim H.W., Park C.W., Shin Y.S., Kim Y.S., Shin S.J., Choi E.J., Chang Y.S., Bang B.K. (2006). Calcitriol regresses cardiac hypertrophy and qt dispersion in secondary hyperparathyroidism on hemodialysis. Nephron Clin. Pract..

[109] Park C.W., Oh Y.S., Shin Y.S., Kim C.M., Kim Y.S., Kim S.Y., Choi E.J., Chang Y.S., Bang B.K. (1999). Intravenous calcitriol regresses myocardial hypertrophy in hemodialysis patients with secondary hyperparathyroidism. Am. J. Kidney Dis..

[110] Xie J., Yoon J., An S.W., Kuro-o M., Huang C.L. (2015). Soluble klotho protects against uremic cardiomyopathy independently of fibroblast growth factor 23 and phosphate. J. Am. Soc. Nephrol..

[111] Schwarz S., Trivedi B.K., Kalantar-Zadeh K., Kovesdy C.P. (2006). Association of disorders in mineral metabolism with progression of chronic kidney disease. Clin. J. Am. Soc. Nephrol..

[112] Block G.A., Klassen P.S., Lazarus J.M., Ofsthun N., Lowrie E.G., Chertow G.M. (2004). Mineral metabolism, mortality and morbidity in maintenance hemodialysis. J. Am. Soc. Nephrol..

[113] Slinin Y., Foley R.N., Collins A.J. (2005). Calcium, phosphorus, parathyroid hormone and cardiovascular disease in hemodialysis patients: The usrds waves 1, 3 and 4 study. J. Am. Soc. Nephrol..

[114] Dhingra R., Sullivan L.M., Fox C.S., Wang T.J., D’Agostino R.B., Gaziano J.M., Vasan R.S. (2007). Relations of serum phosphorus and calcium levels to the incidence of cardiovascular disease in the community. Arch. Intern. Med..

[115] Onufrak S.J., Bellasi A., Cardarelli F., Vaccarino V., Muntner P., Shaw L.J., Raggi P. (2009). Investigation of gender heterogeneity in the associations of serum phosphorus with incident coronary artery disease and all-cause mortality. Am. J. Epidemiol..

[116] Block G.A., Raggi P., Bellasi A., Kooienga L., Spiegel D.M. (2007). Mortality effect of coronary calcification and phosphate binder choice in incident hemodialysis patients. Kidney Int..

[117] Amann K., Tornig J., Kugel B., Gross M.L., Tyralla K., El-Shakmak A., Szabo A., Ritz E. (2003). Hyperphosphatemia aggravates cardiac fibrosis and microvascular disease in experimental uremia. Kidney Int..

[118] Russo D., Corrao S., Battaglia Y., Andreucci M., Caiazza A., Carlomagno A., Lamberti M., Pezone N., Pota A., Russo L. (2011). Progression of coronary artery calcification and cardiac events in patients with chronic renal disease not receiving dialysis. Kidney Int..

[119] Wang S., Qin L., Wu T., Deng B., Sun Y., Hu D., Mohan C., Zhou X.J., Peng A. (2014). Elevated cardiac markers in chronic kidney disease as a consequence of hyperphosphatemia-induced cardiac myocyte injury. Med. Sci. Monit..

[120] Liu Y.L., Lin K.H., Tamilselvi S., Chen W.K., Shen C.Y., Chen R.J., Day C.H., Wu H.C., Viswanadha V.P., Huang C.Y. (2017). Elevated phosphate levels trigger autophagy-mediated cellular apoptosis in h9c2 cardiomyoblasts. Cardiorenal Med..

[121] Yamazaki-Nakazawa A., Mizobuchi M., Ogata H., Kumata C., Kondo F., Ono N., Koiwa F., Uda S., Kinugasa E., Akizawa T. (2014). Correction of hyperphosphatemia suppresses cardiac remodeling in uremic rats. Clin. Exp. Nephrol..

[122] De Boer I.H., Gorodetskaya I., Young B., Hsu C.Y., Chertow G.M. (2002). The severity of secondary hyperparathyroidism in chronic renal insufficiency is gfr-dependent, race-dependent and associated with cardiovascular disease. J. Am. Soc. Nephrol..

[123] Kurosu H., Ogawa Y., Miyoshi M., Yamamoto M., Nandi A., Rosenblatt K.P., Baum M.G., Schiavi S., Hu M.C., Moe O.W. (2006). Regulation of fibroblast growth factor-23 signaling by klotho. J. Biol. Chem..

[124] Galitzer H., Ben-Dov I.Z., Silver J., Naveh-Many T. (2010). Parathyroid cell resistance to fibroblast growth factor 23 in secondary hyperparathyroidism of chronic kidney disease. Kidney Int..

[125] Almaden Y., Canalejo A., Ballesteros E., Anon G., Canadillas S., Rodriguez M. (2002). Regulation of arachidonic acid production by intracellular calcium in parathyroid cells: Effect of extracellular phosphate. J. Am. Soc. Nephrol..

[126] Slatopolsky E., Brown A., Dusso A. (2005). Calcium, phosphorus and vitamin d disorders in uremia. Contrib. Nephrol..

[127] Lopez-Hilker S., Dusso A.S., Rapp N.S., Martin K.J., Slatopolsky E. (1990). Phosphorus restriction reverses hyperparathyroidism in uremia independent of changes in calcium and calcitriol. Am. J. Physiol..

[128] Yumita S., Suzuki M., Akiba T., Akizawa T., Seino Y., Kurokawa K. (1996). Levels of serum 1,25(oh)2d in patients with pre-dialysis chronic renal failure. Tohoku J. Exp. Med..

[129] Fujii H., Kim J.I., Abe T., Umezu M., Fukagawa M. (2007). Relationship between parathyroid hormone and cardiac abnormalities in chronic dialysis patients. Intern. Med..

[130] Ganesh S.K., Stack A.G., Levin N.W., Hulbert-Shearon T., Port F.K. (2001). Association of elevated serum po(4), ca x po(4) product and parathyroid hormone with cardiac mortality risk in chronic hemodialysis patients. J. Am. Soc. Nephrol..

[131] Lishmanov A., Dorairajan S., Pak Y., Chaudhary K., Chockalingam A. (2012). Elevated serum parathyroid hormone is a cardiovascular risk factor in moderate chronic kidney disease. Int. Urol. Nephrol..

[132] Van Ballegooijen A.J., Reinders I., Visser M., Brouwer I.A. (2013). Parathyroid hormone and cardiovascular disease events: A systematic review and meta-analysis of prospective studies. Am. Heart J..

[133] Schluter K.D., Piper H.M. (1998). Cardiovascular actions of parathyroid hormone and parathyroid hormone-related peptide. Cardiovasc. Res..

[134] Harnett J.D., Parfrey P.S., Griffiths S.M., Gault M.H., Barre P., Guttmann R.D. (1988). Left ventricular hypertrophy in end-stage renal disease. Nephron.

[135] Sato S., Ohta M., Kawaguchi Y., Okada H., Ono M., Saito H., Utsunomiya M., Tamura T., Sugimoto K., Takamizawa S. (1995). Effects of parathyroidectomy on left ventricular mass in patients with hyperparathyroidism. Miner. Electrolyte Metab..

[136] Amann K., Ritz E., Wiest G., Klaus G., Mall G. (1994). A role of parathyroid hormone for the activation of cardiac fibroblasts in uremia. J. Am. Soc. Nephrol..

[137] De Loor H., Bammens B., Evenepoel P., De Preter V., Verbeke K. (2005). Gas chromatographic-mass spectrometric analysis for measurement of p-cresol and its conjugated metabolites in uremic and normal serum. Clin. Chem..

[138] Barreto F.C., Barreto D.V., Liabeuf S., Meert N., Glorieux G., Temmar M., Choukroun G., Vanholder R., Massy Z.A. (2009). Serum indoxyl sulfate is associated with vascular disease and mortality in chronic kidney disease patients. Clin. J. Am. Soc. Nephrol..

[139] Liu S., Wang B.H., Kompa A.R., Lekawanvijit S., Krum H. (2012). Antagonists of organic anion transporters 1 and 3 ameliorate adverse cardiac remodelling induced by uremic toxin indoxyl sulfate. Int. J. Cardiol..

[140] Lekawanvijit S., Adrahtas A., Kelly D.J., Kompa A.R., Wang B.H., Krum H. (2010). Does indoxyl sulfate, a uraemic toxin, have direct effects on cardiac fibroblasts and myocytes?. Eur. Heart J..

[141] Lekawanvijit S., Kompa A.R., Manabe M., Wang B.H., Langham R.G., Nishijima F., Kelly D.J., Krum H. (2012). Chronic kidney disease-induced cardiac fibrosis is ameliorated by reducing circulating levels of a non-dialysable uremic toxin, indoxyl sulfate. PLoS ONE.

[142] Fujii H., Nishijima F., Goto S., Sugano M., Yamato H., Kitazawa R., Kitazawa S., Fukagawa M. (2009). Oral charcoal adsorbent (ast-120) prevents progression of cardiac damage in chronic kidney disease through suppression of oxidative stress. Nephrol. Dial. Transpl..

[143] Dou L., Bertrand E., Cerini C., Faure V., Sampol J., Vanholder R., Berland Y., Brunet P. (2004). The uremic solutes p-cresol and indoxyl sulfate inhibit endothelial proliferation and wound repair. Kidney Int..

[144] Dou L., Jourde-Chiche N., Faure V., Cerini C., Berland Y., Dignat-George F., Brunet P. (2007). The uremic solute indoxyl sulfate induces oxidative stress in endothelial cells. J. Thromb. Haemost..

[145] Yu M., Kim Y.J., Kang D.H. (2011). Indoxyl sulfate-induced endothelial dysfunction in patients with chronic kidney disease via an induction of oxidative stress. Clin. J. Am. Soc. Nephrol..

[146] Koizumi M., Tatebe J., Watanabe I., Yamazaki J., Ikeda T., Morita T. (2014). Aryl hydrocarbon receptor mediates indoxyl sulfate-induced cellular senescence in human umbilical vein endothelial cells. J. Atheroscler. Thromb.

[147] Yamamoto H., Tsuruoka S., Ioka T., Ando H., Ito C., Akimoto T., Fujimura A., Asano Y., Kusano E. (2006). Indoxyl sulfate stimulates proliferation of rat vascular smooth muscle cells. Kidney Int..

[148] Muteliefu G., Enomoto A., Jiang P., Takahashi M., Niwa T. (2009). Indoxyl sulphate induces oxidative stress and the expression of osteoblast-specific proteins in vascular smooth muscle cells. Nephrol. Dial. Transpl..

[149] Adelibieke Y., Yisireyili M., Ng H.Y., Saito S., Nishijima F., Niwa T. (2014). Indoxyl sulfate induces il-6 expression in vascular endothelial and smooth muscle cells through oat3-mediated uptake and activation of ahr/nf-kappab pathway. Nephron Exp. Nephrol..

[150] Pletinck A., Glorieux G., Schepers E., Cohen G., Gondouin B., Van Landschoot M., Eloot S., Rops A., Van de Voorde J., De Vriese A. (2013). Protein-bound uremic toxins stimulate crosstalk between leukocytes and vessel wall. J. Am. Soc. Nephrol..

[151] Adijiang A., Higuchi Y., Nishijima F., Shimizu H., Niwa T. (2010). Indoxyl sulfate, a uremic toxin, promotes cell senescence in aorta of hypertensive rats. Biochem. Biophys. Res. Commun..

[152] Chen W.T., Chen Y.C., Hsieh M.H., Huang S.Y., Kao Y.H., Chen Y.A., Lin Y.K., Chen S.A., Chen Y.J. (2015). The uremic toxin indoxyl sulfate increases pulmonary vein and atrial arrhythmogenesis. J. Cardiovasc. Electrophysiol..

[153] Wu I.W., Hsu K.H., Hsu H.J., Lee C.C., Sun C.Y., Tsai C.J., Wu M.S. (2012). Serum free p-cresyl sulfate levels predict cardiovascular and all-cause mortality in elderly hemodialysis patients--a prospective cohort study. Nephrol. Dial. Transpl..

[154] Liabeuf S., Barreto D.V., Barreto F.C., Meert N., Glorieux G., Schepers E., Temmar M., Choukroun G., Vanholder R., Massy Z.A. (2010). Free p-cresylsulphate is a predictor of mortality in patients at different stages of chronic kidney disease. Nephrol. Dial. Transpl..

[155] Wu I.W., Hsu K.H., Lee C.C., Sun C.Y., Hsu H.J., Tsai C.J., Tzen C.Y., Wang Y.C., Lin C.Y., Wu M.S. (2011). P-cresyl sulphate and indoxyl sulphate predict progression of chronic kidney disease. Nephrol. Dial. Transpl..

[156] Lekawanvijit S., Kompa A.R., Wang B.H., Kelly D.J., Krum H. (2012). Cardiorenal syndrome: The emerging role of protein-bound uremic toxins. Circ. Res..

[157] Han H., Zhu J., Zhu Z., Ni J., Du R., Dai Y., Chen Y., Wu Z., Lu L., Zhang R. (2015). P-cresyl sulfate aggravates cardiac dysfunction associated with chronic kidney disease by enhancing apoptosis of cardiomyocytes. J. Am. Heart Assoc..

[158] Meijers B.K., Van Kerckhoven S., Verbeke K., Dehaen W., Vanrenterghem Y., Hoylaerts M.F., Evenepoel P. (2009). The uremic retention solute p-cresyl sulfate and markers of endothelial damage. Am. J. Kidney Dis..

[159] Neirynck N., Vanholder R., Schepers E., Eloot S., Pletinck A., Glorieux G. (2012). An update on uremic toxins. Int. Urol. Nephrol..

[160] Schepers E., Meert N., Glorieux G., Goeman J., Van der Eycken J., Vanholder R. (2007). P-cresylsulphate, the main in vivo metabolite of p-cresol, activates leucocyte free radical production. Nephrol. Dial. Transpl..

[161] Meert N., Schepers E., Glorieux G., Van Landschoot M., Goeman J.L., Waterloos M.A., Dhondt A., Van der Eycken J., Vanholder R. (2011). Novel method for simultaneous determination of p-cresylsulphate and p-cresylglucuronide: Clinical data and pathophysiological implications. Nephrol. Dial. Transpl..

[162] Meijers B.K., Claes K., Bammens B., de Loor H., Viaene L., Verbeke K., Kuypers D., Vanrenterghem Y., Evenepoel P. (2010). P-cresol and cardiovascular risk in mild-to-moderate kidney disease. Clin. J. Am. Soc. Nephrol..

[163] Bammens B., Evenepoel P., Keuleers H., Verbeke K., Vanrenterghem Y. (2006). Free serum concentrations of the protein-bound retention solute p-cresol predict mortality in hemodialysis patients. Kidney Int..

[164] Peng Y.S., Ding H.C., Lin Y.T., Syu J.P., Chen Y., Wang S.M. (2012). Uremic toxin p-cresol induces disassembly of gap junctions of cardiomyocytes. Toxicology.

[165] Dou L., Cerini C., Brunet P., Guilianelli C., Moal V., Grau G., De Smet R., Vanholder R., Sampol J., Berland Y. (2002). P-cresol, a uremic toxin, decreases endothelial cell response to inflammatory cytokines. Kidney Int..

[166] Dou L., Sallee M., Cerini C., Poitevin S., Gondouin B., Jourde-Chiche N., Fallague K., Brunet P., Calaf R., Dussol B. (2014). The cardiovascular effect of the uremic solute indole-3 acetic acid. J. Am. Soc. Nephrol..

[167] Jourde-Chiche N., Dou L., Sabatier F., Calaf R., Cerini C., Robert S., Camoin-Jau L., Charpiot P., Argiles A., Dignat-George F. (2009). Levels of circulating endothelial progenitor cells are related to uremic toxins and vascular injury in hemodialysis patients. J. Thromb Haemost..

[168] Moustapha A., Naso A., Nahlawi M., Gupta A., Arheart K.L., Jacobsen D.W., Robinson K., Dennis V.W. (1998). Prospective study of hyperhomocysteinemia as an adverse cardiovascular risk factor in end-stage renal disease. Circulation.

[169] Wald D.S., Law M., Morris J.K. (2002). Homocysteine and cardiovascular disease: Evidence on causality from a meta-analysis. BMJ.

[170] Heinz J., Kropf S., Luley C., Dierkes J. (2009). Homocysteine as a risk factor for cardiovascular disease in patients treated by dialysis: A meta-analysis. Am. J. Kidney Dis..

[171] Jardine M.J., Kang A., Zoungas S., Navaneethan S.D., Ninomiya T., Nigwekar S.U., Gallagher M.P., Cass A., Strippoli G., Perkovic V. (2012). The effect of folic acid based homocysteine lowering on cardiovascular events in people with kidney disease: Systematic review and meta-analysis. BMJ.

[172] Brown J.C., Rosenquist T.H., Monaghan D.T. (1998). Erk2 activation by homocysteine in vascular smooth muscle cells. Biochem. Biophys. Res. Commun..

[173] Van Campenhout A., Moran C.S., Parr A., Clancy P., Rush C., Jakubowski H., Golledge J. (2009). Role of homocysteine in aortic calcification and osteogenic cell differentiation. Atherosclerosis.

[174] Hofmann M.A., Lalla E., Lu Y., Gleason M.R., Wolf B.M., Tanji N., Ferran L.J., Kohl B., Rao V., Kisiel W. (2001). Hyperhomocysteinemia enhances vascular inflammation and accelerates atherosclerosis in a murine model. J. Clin. Investig..

[175] Lee J.C., Downing S.E. (1981). Negative inotropic effects of phenol on isolated cardiac muscle. Am. J. Pathol..

[176] Sharma A., Patil J.A., Gramajo A.L., Seigel G.M., Kuppermann B.D., Kenney C.M. (2012). Effects of hydroquinone on retinal and vascular cells in vitro. Indian J. Ophthalmol..

[177] Eloot S., Schneditz D., Cornelis T., Van Biesen W., Glorieux G., Dhondt A., Kooman J., Vanholder R. (2016). Protein-bound uremic toxin profiling as a tool to optimize hemodialysis. PLoS ONE.

[178] Nishio T., Takamura N., Nishii R., Tokunaga J., Yoshimoto M., Kawai K. (2008). Influences of haemodialysis on the binding sites of human serum albumin: Possibility of an efficacious administration plan using binding inhibition. Nephrol. Dial. Transpl..

[179] Lesaffer G., De Smet R., Lameire N., Dhondt A., Duym P., Vanholder R. (2000). Intradialytic removal of protein-bound uraemic toxins: Role of solute characteristics and of dialyser membrane. Nephrol. Dial. Transpl..

[180] Thambyrajah J., Townend J.N. (2000). Homocysteine and atherothrombosis—Mechanisms for injury. Eur. Heart J..

[181] Yang K., Xu X., Nie L., Xiao T., Guan X., He T., Yu Y., Liu L., Huang Y., Zhang J. (2015). Indoxyl sulfate induces oxidative stress and hypertrophy in cardiomyocytes by inhibiting the ampk/ucp2 signaling pathway. Toxicol. Lett..

[182] Heath-Pagliuso S., Rogers W.J., Tullis K., Seidel S.D., Cenijn P.H., Brouwer A., Denison M.S. (1998). Activation of the ah receptor by tryptophan and tryptophan metabolites. Biochemistry.

[183] Schroeder J.C., Dinatale B.C., Murray I.A., Flaveny C.A., Liu Q., Laurenzana E.M., Lin J.M., Strom S.C., Omiecinski C.J., Amin S. (2010). The uremic toxin 3-indoxyl sulfate is a potent endogenous agonist for the human aryl hydrocarbon receptor. Biochemistry.

[184] Watanabe I., Tatebe J., Namba S., Koizumi M., Yamazaki J., Morita T. (2013). Activation of aryl hydrocarbon receptor mediates indoxyl sulfate-induced monocyte chemoattractant protein-1 expression in human umbilical vein endothelial cells. Circ. J..

[185] Ito S., Osaka M., Edamatsu T., Itoh Y., Yoshida M. (2016). Crucial role of the aryl hydrocarbon receptor (ahr) in indoxyl sulfate-induced vascular inflammation. J. Atheroscler. Thromb.

[186] Gondouin B., Cerini C., Dou L., Sallee M., Duval-Sabatier A., Pletinck A., Calaf R., Lacroix R., Jourde-Chiche N., Poitevin S. (2013). Indolic uremic solutes increase tissue factor production in endothelial cells by the aryl hydrocarbon receptor pathway. Kidney Int..

[187] Carney S.A., Chen J., Burns C.G., Xiong K.M., Peterson R.E., Heideman W. (2006). Aryl hydrocarbon receptor activation produces heart-specific transcriptional and toxic responses in developing zebrafish. Mol. Pharmacol..

[188] Yang K., Nie L., Huang Y., Zhang J., Xiao T., Guan X., Zhao J. (2012). Amelioration of uremic toxin indoxyl sulfate-induced endothelial cell dysfunction by klotho protein. Toxicol Lett..

[189] Sun C.Y., Chang S.C., Wu M.S. (2012). Suppression of klotho expression by protein-bound uremic toxins is associated with increased DNA methyltransferase expression and DNA hypermethylation. Kidney Int..

[190] Adijiang A., Niwa T. (2010). An oral sorbent, ast-120, increases klotho expression and inhibits cell senescence in the kidney of uremic rats. Am. J. Nephrol..

[191] Shimizu H., Bolati D., Adijiang A., Adelibieke Y., Muteliefu G., Enomoto A., Higashiyama Y., Higuchi Y., Nishijima F., Niwa T. (2011). Indoxyl sulfate downregulates renal expression of klotho through production of ros and activation of nuclear factor-kb. Am. J. Nephrol..

[192] Corsetti G., Pasini E., Scarabelli T.M., Romano C., Agrawal P.R., Chen-Scarabelli C., Knight R., Saravolatz L., Narula J., Ferrari-Vivaldi M. (2016). Decreased expression of klotho in cardiac atria biopsy samples from patients at higher risk of atherosclerotic cardiovascular disease. J. Geriatr. Cardiol..

[193] Atoh K., Itoh H., Haneda M. (2009). Serum indoxyl sulfate levels in patients with diabetic nephropathy: Relation to renal function. Diabetes Res. Clin. Pr..

[194] Evenepoel P., Meijers B.K., Bammens B.R., Verbeke K. (2009). Uremic toxins originating from colonic microbial metabolism. Kidney Int. Suppl..

[195] Marzocco S., Dal Piaz F., Di Micco L., Torraca S., Sirico M.L., Tartaglia D., Autore G., Di Iorio B. (2013). Very low protein diet reduces indoxyl sulfate levels in chronic kidney disease. Blood Purif..

[196] Hida M., Aiba Y., Sawamura S., Suzuki N., Satoh T., Koga Y. (1996). Inhibition of the accumulation of uremic toxins in the blood and their precursors in the feces after oral administration of lebenin, a lactic acid bacteria preparation, to uremic patients undergoing hemodialysis. Nephron.

[197] Takayama F., Taki K., Niwa T. (2003). Bifidobacterium in gastro-resistant seamless capsule reduces serum levels of indoxyl sulfate in patients on hemodialysis. Am. J. Kidney Dis..

[198] Furuse S.U., Ohse T., Jo-Watanabe A., Shigehisa A., Kawakami K., Matsuki T., Chonan O., Nangaku M. (2014). Galacto-oligosaccharides attenuate renal injury with microbiota modification. Physiol. Rep..

[199] Nakabayashi I., Nakamura M., Kawakami K., Ohta T., Kato I., Uchida K., Yoshida M. (2011). Effects of synbiotic treatment on serum level of p-cresol in haemodialysis patients: A preliminary study. Nephrol. Dial. Transpl..

[200] Guida B., Germano R., Trio R., Russo D., Memoli B., Grumetto L., Barbato F., Cataldi M. (2014). Effect of short-term synbiotic treatment on plasma p-cresol levels in patients with chronic renal failure: A randomized clinical trial. Nutr. Metab. Cardiovasc. Dis..

[201] Mishima E., Fukuda S., Shima H., Hirayama A., Akiyama Y., Takeuchi Y., Fukuda N.N., Suzuki T., Suzuki C., Yuri A. (2014). Alteration of the intestinal environment by lubiprostone is associated with amelioration of adenine-induced ckd. J. Am. Soc. Nephrol..

[202] Shen B., Pardi D.S., Bennett A.E., Queener E., Kammer P., Hammel J.P., LaPlaca C., Harris M.S. (2009). The efficacy and tolerability of ast-120 (spherical carbon adsorbent) in active pouchitis. Am. J. Gastroenterol..

[203] Aoyama I., Shimokata K., Niwa T. (2002). An oral adsorbent downregulates renal expression of genes that promote interstitial inflammation and fibrosis in diabetic rats. Nephron.

[204] Niwa T., Ise M. (1994). Indoxyl sulfate, a circulating uremic toxin, stimulates the progression of glomerular sclerosis. J. Lab. Clin. Med..

[205] Nakagawa N., Hasebe N., Sumitomo K., Fujino T., Fukuzawa J., Hirayama T., Kikuchi K. (2006). An oral adsorbent, ast-120, suppresses oxidative stress in uremic rats. Am. J. Nephrol..

[206] Yamamoto S., Zuo Y., Ma J., Yancey P.G., Hunley T.E., Motojima M., Fogo A.B., Linton M.F., Fazio S., Ichikawa I. (2011). Oral activated charcoal adsorbent (ast-120) ameliorates extent and instability of atherosclerosis accelerated by kidney disease in apolipoprotein e-deficient mice. Nephrol. Dial. Transpl..

[207] Sato E., Saigusa D., Mishima E., Uchida T., Miura D., Morikawa-Ichinose T., Kisu K., Sekimoto A., Saito R., Oe Y. (2018). Impact of the oral adsorbent ast-120 on organ-specific accumulation of uremic toxins: Lc-ms/ms and ms imaging techniques. Toxins.

[208] Akizawa T., Asano Y., Morita S., Wakita T., Onishi Y., Fukuhara S., Gejyo F., Matsuo S., Yorioka N., Kurokawa K. (2009). Effect of a carbonaceous oral adsorbent on the progression of ckd: A multicenter, randomized, controlled trial. Am. J. Kidney Dis..

[209] Schulman G., Berl T., Beck G.J., Remuzzi G., Ritz E., Arita K., Kato A., Shimizu M. (2014). Randomized placebo-controlled eppic trials of ast-120 in ckd. J. Am. Soc. Nephrol..

